# Impact of Dietary Resistant Starch on the Human Gut Microbiome, Metaproteome, and Metabolome

**DOI:** 10.1128/mBio.01343-17

**Published:** 2017-10-17

**Authors:** Tanja V. Maier, Marianna Lucio, Lang Ho Lee, Nathan C. VerBerkmoes, Colin J. Brislawn, Jörg Bernhardt, Regina Lamendella, Jason E. McDermott, Nathalie Bergeron, Silke S. Heinzmann, James T. Morton, Antonio González, Gail Ackermann, Rob Knight, Katharina Riedel, Ronald M. Krauss, Philippe Schmitt-Kopplin, Janet K. Jansson

**Affiliations:** a Research Unit Analytical BioGeoChemistry, Helmholtz Zentrum München, Neuherberg, Germany; b Center for Interdisciplinary Cardiovascular Science (CICS), Brigham and Women's Hospital and Harvard Medical School, Boston, Massachusetts, USA; c The University of Texas, El Paso, Texas, USA; d Earth and Biological Sciences Directorate, Pacific Northwest National Laboratory, Richland, Washington, USA; e Institute of Microbiology, Greifswald University, Greifswald, Germany; f Juniata College, Huntingdon, Pennsylvania, USA; g Department of Molecular Microbiology and Immunology, Oregon Health & Sciences University, Portland, Oregon, USA; h Children’s Hospital Oakland Research Institute, Oakland, California, USA; i College of Pharmacy, Touro University California, Vallejo, California, USA; j University of California, San Diego, California, USA; k Technische Universität München, Freising, Germany; University of Georgia

**Keywords:** gut microbiome, human microbiome, multiomics, resistant starch

## Abstract

Diet can influence the composition of the human microbiome, and yet relatively few dietary ingredients have been systematically investigated with respect to their impact on the functional potential of the microbiome. Dietary resistant starch (RS) has been shown to have health benefits, but we lack a mechanistic understanding of the metabolic processes that occur in the gut during digestion of RS. Here, we collected samples during a dietary crossover study with diets containing large or small amounts of RS. We determined the impact of RS on the gut microbiome and metabolic pathways in the gut, using a combination of “omics” approaches, including 16S rRNA gene sequencing, metaproteomics, and metabolomics. This multiomics approach captured changes in the abundance of specific bacterial species, proteins, and metabolites after a diet high in resistant starch (HRS), providing key insights into the influence of dietary interventions on the gut microbiome. The combined data showed that a high-RS diet caused an increase in the ratio of *Firmicutes* to *Bacteroidetes*, including increases in relative abundances of some specific members of the *Firmicutes* and concurrent increases in enzymatic pathways and metabolites involved in lipid metabolism in the gut.

## IMPORTANCE

This work was undertaken to obtain a mechanistic understanding of the complex interplay between diet and the microorganisms residing in the intestine. Although it is known that gut microbes play a key role in digestion of the food that we consume, the specific contributions of different microorganisms are not well understood. In addition, the metabolic pathways and resultant products of metabolism during digestion are highly complex. To address these knowledge gaps, we used a combination of molecular approaches to determine the identities of the microorganisms in the gut during digestion of dietary starch as well as the metabolic pathways that they carry out. Together, these data provide a more complete picture of the function of the gut microbiome in digestion, including links between an RS diet and lipid metabolism and novel linkages between specific gut microbes and their metabolites and proteins produced in the gut.

## INTRODUCTION

Prebiotics include some classes of dietary carbohydrates that are resistant to degradation in the small intestine but metabolized by microbes in the colon, where they are fermented into short-chain fatty acids (SCFA), gases, and other products, which directly or indirectly affect the health of the host (1). The amount and types of carbohydrates that reach the colon affect the composition of the gut microbiome (1, 2) as well as the metabolic end products of microbial degradation (3). Foods that are enriched with resistant but fermentable starches are of interest as prebiotics due to their potential health benefits.

Resistant starch (RS) is an example of a complex carbohydrate and prebiotic that is relatively resistant to degradation in the small intestine by α-amylase, a starch degradation enzyme produced by the host. The degree of resistance to degradation is largely dependent on the proportion of amylose to amylopectin that the starch molecule contains. Amylopectin is a glucose polymer that is susceptible to enzymatic hydrolysis by amylase at branching points occurring at α1-6 glycosidic bonds every 24 to 30 glucose units. In contrast, amylose is a more linear glucose polymer with primarily α1-4 glycosidic bonds that are not easily hydrolyzed. The crystallinity, particle size, structure, and cooking approach are also factors that contribute to the digestibility of starches in the diet. In this respect, RS can be classified into 4 different types: RS type 1 is physically inaccessible, RS type 2 is native granular starch consisting of ungelatinized granules, RS type 3 is retrograded amylose, and RS type 4 is chemically modified to make it indigestible. Depending on the formulation of RS, the gut microbiome has been reported to respond differently, with a trend for an increase in *Bacteroidetes* compared to *Firmicutes* after an RS4 diet (4) and the opposite trend after an RS2 diet (5).

Once RS enters the colon in a form that is accessible to microbial digestion, it is fermented to SCFA, such as butyrate. Butyrate has several proposed health benefits, including provision of energy for colonic epithelial cells and improvement of insulin sensitivity (6). Previous studies have established links between specific members of the gut microbiome and RS digestion (2), including the key role of some members in fermentation of RS to butyrate (7, 8). Specific taxa that have been shown to be involved in RS metabolism include *Faecalibacterium prausnitzii*, *Eubacterium rectale*, and *Ruminococcus bromii* (2, 9–11).

Here, we aimed to go beyond understanding impacts of RS at the microbial community level, to gain a more complete understanding at a mechanistic level of the impact of RS on the metabolic functions that are carried out by members of the gut microbiome during RS digestion in conjunction with the host. We studied the gut microbiome in stool samples collected from a cohort of individuals who had a prescribed diet with high or low levels of RS in a crossover study design. We used a within-subject crossover design to determine at what levels the responses were most clearly manifested. To identify the functional potential of the gut microbiome during RS digestion, we leveraged our development of untargeted “shotgun” approaches to determine the complement of proteins (metaproteomics) (12) and metabolites (13) in the gut. Together, this multiomics approach enabled us to develop a more complete picture of the metabolic processes occurring in the gut during RS digestion.

## RESULTS AND DISCUSSION

The dietary study included 39 participants with reduced insulin sensitivity, as assessed using a homeostatic model assessment of insulin resistance (HOMA-IR) below the median (14). The rationale for choosing insulin-resistant subjects was to determine whether the diet would improve insulin sensitivity, as previously reported (15–18). The participants consumed diets with either large amounts of carbohydrates (*n* = 16) or small amounts of carbohydrates (*n* = 23), following a baseline diet. Next, all participants consumed either large or small amounts of resistant starch (HRS or LRS, respectively) for 2 weeks, in a crossover time series study (see Fig. S1A in the supplemental material), with a 2-week baseline washout diet in between. Fecal samples were collected after the baseline diet (day 14) and again after the LRS and HRS diets (day 28 or day 56, respectively), for a total of 3 samples per subject (Fig. S1A and B). Macronutrient distributions in the baseline diet and both RS diets were similar, whereas the baseline diet was low in foods containing naturally occurring RS. The HRS diet included high-amylose cornstarch (Hi-Maize 260; Ingredion Inc., Bridgewater, NJ; 41.5 g RS/100 g starch), while the LRS diet contained conventional, high-amylopectin cornstarch (Melojel; Ingredion Inc., Bridgewater, NJ; 2.3 g RS/100 g starch). The amount of RS was designed to match the carbohydrate load of the diet. Therefore, subjects in the high-carbohydrate (HC) arm of the study consumed either 66 g RS for the HRS diet or 4 g for the LRS diet, whereas for the low-carbohydrate (LC) arm of the study the subjects consumed either 48 g for the HRS diet or 3 g for the LRS diet, based on 2500 kcal/day menus (Fig. S1B). In the LRS diet group, Melojel cornstarch was consumed cooked and in baked goods, while approximately 50% of the Hi-Maize cornstarch in the HRS diet group was consumed raw and mixed with beverages, soups, or fruit purees. The HRS and LRS diets were otherwise balanced with respect to amounts of fat, protein, and food fiber, as described elsewhere (19).

10.1128/mBio.01343-17.1 FIG S1  Crossover study design. Download FIG S1, PDF file, 0.4 MB. Copyright © 2017 Maier et al. 2017 Maier et al. This content is distributed under the terms of the Creative Commons Attribution 4.0 International license.

All of the fecal samples were subjected to 16S rRNA gene (16S) sequencing to determine the impact of diet on the gut microbial community structure. Interpersonal variation was identified as an important factor, with samples clustering by patient throughout the dietary intervention (Fig. S2A). The effect of prescribed diet was also significant, and the low-carbohydrate diet showed the greatest impact of RS supplementation on the microbial community structure (Fig. S2B). Therefore, the remaining analyses that we present here focus on the samples from the low-carbohydrate diet arm of the study (23 participants; 3 samples taken at 3 time points each, for a total of 69 fecal samples [Fig. S1B]). The 16S data also revealed that diet had a significant impact on the microbiome structure, irrespective of the time of sampling during the crossover study. For this reason, we classified samples based on the resistant starch load of the diet at the time of sampling (baseline, HRS, or LRS, respectively), without differentiating if that diet was assigned first or second during the crossover study.

10.1128/mBio.01343-17.2 FIG S2  Principal coordinate analysis (PCoA) ordination of unweighted UniFrac distances between 16S rRNA gene sequences for each stool sample. Download FIG S2, PDF file, 0.5 MB. Copyright © 2017 Maier et al. 2017 Maier et al. This content is distributed under the terms of the Creative Commons Attribution 4.0 International license.

In agreement with earlier observations (15–18), we reported significantly attenuated postprandial insulin and glucose responses to the HRS meals (19). Whereas the HRS and LRS diets did not affect fasting concentrations of insulin and glucose, the HRS meals (19) produced significantly lower postprandial insulin and glucose responses, expressed as incremental area under the curve (IAUC), compared to LRS meals (19). These results suggest a potential utility for RS in improving meal-to-meal regulation of blood glucose. However, we also found that plasma levels of trimethylamine-*N*-oxide (TMAO), a biomarker of cardiovascular disease (CVD) risk, were higher following the HRS diet (19). Therefore, the relative benefits of dietary RS should be further investigated, probably on a per-individual basis.

**FIG 1:**
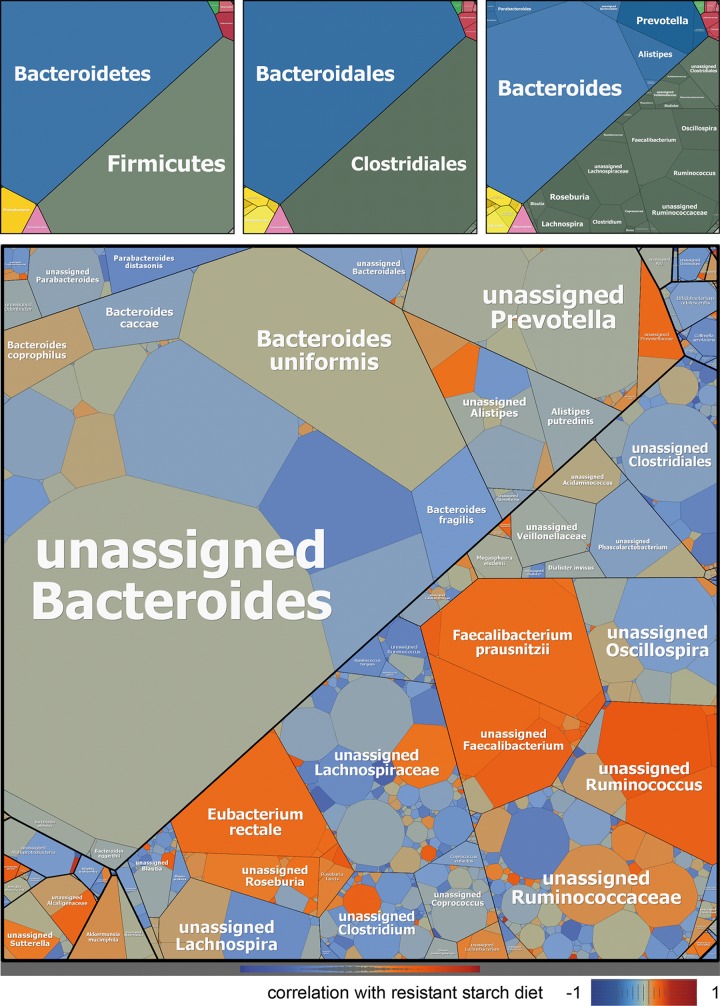
Map of the microbiome corresponding to dietary resistant starch (RS) levels. Average relative abundances of taxa are based on 16S rRNA gene sequences from all samples: the larger the cells, the greater the overall abundance of operational taxonomic units (OTUs) in that particular taxonomic category averaged across all samples in this study. Maps on top from left to right specify phylum, order, and genus. The map on the bottom visualizes averaged Pearson correlation coefficients from all samples that were calculated from relative OTU abundances versus the approximate amount of RS in each diet: 0 (baseline), 0.05 (LRS), and 1 (HRS).

Consistent with previous reports (2), we found that the HRS diet resulted in a shift in the structure of the gut microbiome (Fig. 1). Although the bacterial structure of the fecal samples varied between individuals (Fig. S3), there was a consistent increase in the proportion of *Firmicutes* to *Bacteroides* following the HRS diet compared to the baseline and LRS diets (Fig. 1), suggesting that members of the *Firmicutes* had a selective advantage over members of the *Bacteroides* when there were large amounts of RS in the diet. These changes included increases in relative amounts of species in the genera *Faecalibacterium*, *Roseburia*, and *Ruminococcus*, which have been associated with butyrate production (20, 21) and found to be reduced in abundance in the gut microbiota of participants with type 2 diabetes mellitus (T2DM) compared to healthy individuals (22). Consistent with the increase of *F. prausnitzii*, *Roseburia*, and *Ruminococcus*, short-chain fatty acid analysis revealed a slight increase of butyrate and propionate in the fecal samples of participants consuming the HRS diet. Valerate and isovalerate were not affected by the different diets. Further, specific taxa that increased following the HRS diet included *Faecalibacterium prausnitzii*, *Prevotellaceae*, *Ruminococcus*, *Eubacterium rectale*, *Roseburia faecis*, and *Akkermansia muciniphila* (Fig. 1), several of which have previously been reported to increase in the colon following a high resistant starch diet (2, 9, 11).

10.1128/mBio.01343-17.3 FIG S3  Individual response to resistant starch. Download FIG S3, PDF file, 1.8 MB. Copyright © 2017 Maier et al. 2017 Maier et al. This content is distributed under the terms of the Creative Commons Attribution 4.0 International license.

**FIG 2:**
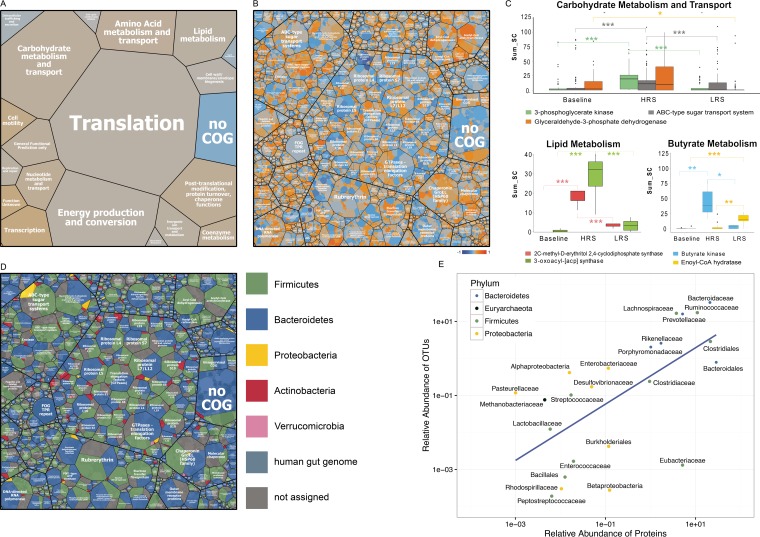
Map of the metaproteome corresponding to dietary resistant starch (RS) levels and assignment of proteins to bacterial phyla. (A) Functional assignments (Clusters of Orthologous Groups [COGs]) of all bacterial proteins across all samples. Cell sizes correspond to averaged protein abundances. (B) Protein functions are shown at a more detailed level. For panels A and B, averaged Pearson correlation coefficients from all individuals were calculated from relative protein abundances versus the approximate amount of RS in each diet: 0 (baseline), 0.05 (LRS), and 1 (HRS). The color scale for the Pearson correlation coefficients is as follows: −1, dark blue, negatively correlated with resistant starch concentration; 0, gray; 1, orange, positively correlated with resistant starch concentration. (C) Examples of specific proteins that significantly differed according to diet (*post hoc* Kruskal-Nemenyi test; *, *P* < 0.05; **, *P* < 0.01; ***, *P* < 0.001). (D) Bacterial taxa that were assigned to the same proteins as shown in panel B were partitioned and color coded according to bacterial phyla. (E) Correlation of the most common OTUs and corresponding proteins, labeled at the family level and colored at phylum level.

In the present study, we went beyond taxonomic characterization to also investigate the functional shifts according to diet. We first employed a shotgun metaproteomics approach (*n* = 24) (12) to determine the identities of thousands of host and microbial proteins across the samples. The main Clusters of Orthologous Groups (COG) classes (Fig. 2A) represented in the protein data included those for translation, carbohydrate metabolism and transport, energy production and conversion, amino acid metabolism and transport, and lipid metabolism. We added taxonomic annotations to the 56,294 bacterial proteins detected (Fig. 2B) and focused on proteins involved in carbohydrate metabolism and transport that were significantly shifted in relative abundance by diet (Fig. 2C). Of these, several proteins involved in butyrate metabolism were significantly altered, as verified with the *post hoc* Kruskal-Nemenyi test (Fig. 2C), including butyrate kinase (baseline versus HRS, *P* < 0.001; HRS versus LRS, *P* < 0.01) and enoyl coenzyme A (enoyl-CoA) hydratase (LRS versus baseline, *P* < 0.0001; LRS versus HRS, *P* < 0.003). A targeted quantification of butyrate in the samples revealed trends for increased butyrate accumulation in the HRS diet and to a lesser extent in the LRS diet, although this was highly variable between individuals (data not shown). Cross-feeding effects between gut microbial populations have previously been shown to increase variability between individuals because butyrate producers often take longer to become established after a dietary intervention (23). Furthermore, proteins involved in energy production and conversion (phosphotransacetylase) and nucleotide metabolism and transport (adenylosuccinate synthase, adenine/guanine phosphoribosyltransferases [PRPPs], and related PRPP-binding proteins) were correlated with the HRS diet compared to baseline (Fig. 2A and B). Proteins assigned to the main COG classes and their corresponding phyla are shown in Fig. 2D; additional significant COG terms are visualized in a heat map in Fig. S4.

10.1128/mBio.01343-17.4 FIG S4  Proteins that vary significantly between diets. Download FIG S4, PDF file, 0.4 MB. Copyright © 2017 Maier et al. 2017 Maier et al. This content is distributed under the terms of the Creative Commons Attribution 4.0 International license.

The use of 16S data together with proteomics provided us with greater resolution of functional roles of members of the gut microbiome. There was a high correlation between the 16S data and the proteome data with respect to operational taxonomic unit (OTU) and protein abundance (Fig. 2E), with more proteins detected for the more abundant community members. For example, some members of the *Firmicutes* and *Bacteroidetes* were both highly abundant at the 16S level and had the largest amount of proteins detected. The most abundant families with the major protein identifications were *Bacteroidaceae*, *Ruminococcaceae*, *Lachnospiraceae*, and *Prevotellaceae*. There were some outliers, e.g., the *Eubacteriaceae*, which had a relatively low abundance but a high number of proteins detected, indicating a high level of protein production per individual in this group. Another outlier was *Pasteurellaceae*, which were relatively abundant but had few proteins assigned, suggesting that the represented populations were not very active at the sampling time. The *Proteobacteria* were intermediate in abundance and protein levels. Furthermore, the taxonomic assignment was attained for many of the proteins (14%) based on the gut reference isolate database used for the proteome searches. Most of the carbohydrate metabolism enzymes and transport systems associated with the HRS diet were affiliated with specific species, such as *F. prausnitzii* and *Coprococcus comes*, some of which were also more abundant following the HRS diet than at baseline and after the LRS diet.

Analysis of human proteins in the samples revealed a relative enrichment of some human proteins involved in lipid metabolism with the HRS diet by comparison to baseline. These included lipases, such as colipase, pancreatic triglyceride lipase, and bile salt-stimulated lipase. In contrast, human α-amylase was negatively correlated with the HRS diet, presumably because of the resistant nature of the starch that made it less accessible as a substrate during digestion (24, 25).

We used high-resolution mass spectrometry (Fourier transform ion cyclotron resonance mass spectrometry [FT-ICR-MS]) to analyze metabolites in the same fecal samples (*n* = 45). Metabolites were extracted with methanol and analyzed by FT-ICR-MS. Data filtering and metabolite assignment using the MassTRIX (26) web server revealed 5,552 features, the majority (62%, 3,416 features) of which were unknown but assigned to molecular formulas using NetCalc (27). Thirty-eight percent (2,136 features) could be assigned to compounds. Nine percent (525 features) of the total 5,552 features are listed in the Kyoto Encyclopedia of Genes and Genomes (KEGG) (28) pathways.

**FIG 3:**
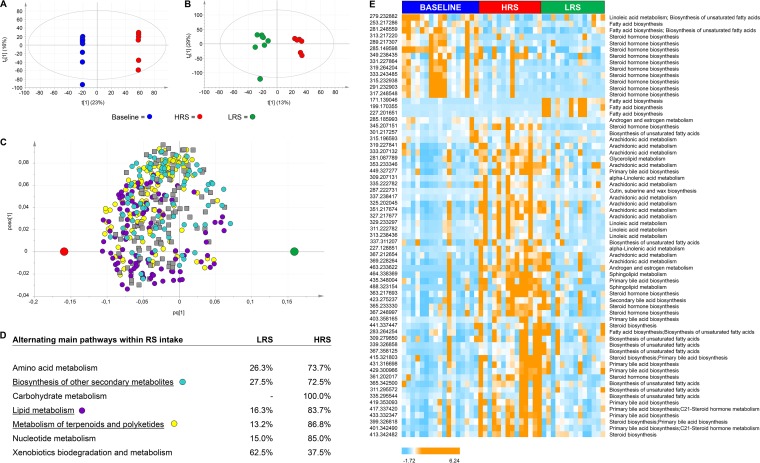
Significant differences in fecal metabolite compositions between diets. (A) OPLS-DA score scatter plot comparing baseline diet (blue) versus HRS diet (red); *Q*²(cum) = 0.8 and *R*^2^*Y*(cum) = 1. (B) OPLS-DA score scatter plot comparing LRS diet (green) with HRS diet (red); *Q*²(cum) = 0.6 and *R*^2^*Y*(cum) = 0.9. (A and B) t[1] represents the first component; t0[1] expresses the variance orthogonal to the variable *Y* (class). (C) OPLS-DA loading scatter plot of metabolites assigned to biosynthesis of other secondary metabolites (cyan), lipid metabolism (purple), and metabolism of terpenoids and polyketides (yellow). (D) Alternating main pathways within different diets (HRS versus LRS); *Q*²(cum) = 0.6 and *R*^2^*Y*(cum) = 0.3. (E) Euclidean distance hierarchical clustering analysis visualizing the different intensity levels of compounds related to lipid metabolism related in specific diet classes.

By application of multivariate statistical analyses (Fig. 3), significant changes in gut metabolites were found between samples collected after the HRS and baseline diets (Fig. 3A) but not between baseline and LRS diets. Furthermore, a trend toward separation was observed between the HRS and LRS diets (Fig. 3B). Several orthogonal partial least-squares discriminant analysis (OPLS-DA) models were applied (Table 1), which revealed significant features that were highly affected by the HRS diet compared to baseline and LRS diets. This showed that almost half (46%, 2,566 features) of the total 5,552 features were altered by diet. As a consequence, 2.7% or 3.0% of the 46% significant features detected were related to the baseline or LRS diets, respectively, whereas a much higher percentage was related to the HRS diet (74.7% of the total significant 2,566 features). Given the high number of unknown metabolites and the accompanying difficulty of identification and classification (29, 30), we focused only on the metabolites that were altered by diet according to OPLS-DA results.

**TABLE 1:** OPLS-DA models for metabolomics analysis

Description	Model	*R*^2^*Y*(cum)	*Q*^2^(cum)	*P* (CV-ANOVA)
Baseline (28 days) versus LRS (28 days), fatty acyls only	G1 versus G3, fatty acyls only	0.881	0.563	0.007
Baseline versus HRS (28 days)	G1 versus G2	0.678	0.521	0.037
Baseline versus HRS (28 days), fatty acyls only	G1 versus G2, fatty acyls only	0.995	0.853	0.00004
Baseline versus LRS (28 days)	G1 versus G3			NS^a^
Baseline versus rest^b^	G1 versus rest	0.835	0.459	0.0005
HRS (28 days) versus HRS (56 days)	G2 versus G5			NS
HRS (28 days) versus LRS (28 days), fatty acyls only	G2 versus G3, fatty acyls only	0.749	0.48	0.027
HRS (28 days) versus LRS (56 days)	G2 versus G4	0.777	0.503	0.043
HRS versus LRS	(G2, G5) versus (G3, G4)	0.443	0.277	0.017
HRS versus rest	(G2, G5) versus rest	0.432	0.324	0.0003
LRS (28 days) versus LRS (56 days)	G3 versus G4			NS
LRS versus HRS (28 days)	(G3, G4) versus G2	0.92	0.56	0.016
LRS versus rest	(G3, G4) versus rest	0.833	0.397	0.014

a NS, not significant.

b Rest, the rest of the diet types.

The metabolite data strengthened the overall evidence that lipid metabolic pathways carried out by both the host and the microbiome were impacted by diet (Fig. 3A and B). Several metabolites in pathways involved in lipid metabolism were significantly higher or lower in abundance in the HRS diet than at baseline (Fig. 3C, D, and E); these included pathways for fatty acid metabolism, primary and secondary bile acid biosynthesis, bile acid secretion, steroid biosynthesis, and metabolism of linoleic and arachidonic acid. In addition, many fatty acids varying in chain length and saturation degrees from C_17_ to C_29_ (Fig. S5) show a positive correlation with the HRS and LRS profiles (*P* < 0.05). Of these, three fatty acids, namely, hexadecanoic acid (C_16:1_), octadecadienoic acid (C_18:2_), and octadecenoic acid (C_18:1_), were less abundant after diets containing high or low degrees of RS (Fig. S5). These novel findings suggest that products of dietary RS fermentation in the colon are further metabolized by the gut microbiome and involved in lipid biosynthesis as well as metabolism of host-derived lipids.

10.1128/mBio.01343-17.5 FIG S5  Classes of fatty acyls grouped according to their relative abundance following a specific diet category. Download FIG S5, PDF file, 0.2 MB. Copyright © 2017 Maier et al. 2017 Maier et al. This content is distributed under the terms of the Creative Commons Attribution 4.0 International license.

Although the baseline and LRS diets were hard to discriminate at the whole-profile level, three features (*m/z* 171.13906, *m/z* 199.17035, and *m/z* 227.20166; C_10:0_, C_12:0_, and C_14:0_, respectively), of which the fatty acids C_12:0_ and C_14:0_ were confirmed by ultrahigh-performance liquid chromatography–mass spectrometry (UHPLC-MS), were significantly (*P* < 0.002) elevated on the LRS diet (Fig. 3E, S5, and S6). These were correlated with a *Lachnospiraceae* species that was also higher in relative abundance after the LRS diet. Further analysis of the center log-transformed metabolomics data revealed that the separation between baseline samples from the other samples was largely explained by abundances of fatty acids and sterol lipids (Fig. S7).

10.1128/mBio.01343-17.6 FIG S6  Identification of decanoic (C_12:0_) and tetradecanoic acid (C_14:0_). Download FIG S6, PDF file, 0.3 MB. Copyright © 2017 Maier et al. 2017 Maier et al. This content is distributed under the terms of the Creative Commons Attribution 4.0 International license.

10.1128/mBio.01343-17.7 FIG S7  Biplot of different metabolite classes on centered, log-transformed data. Download FIG S7, PDF file, 0.7 MB. Copyright © 2017 Maier et al. 2017 Maier et al. This content is distributed under the terms of the Creative Commons Attribution 4.0 International license.

**FIG 4:**
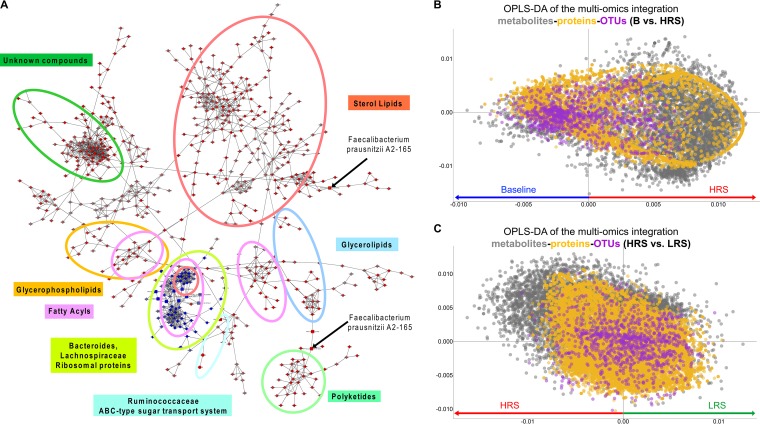
Multiomics data integration for different diet categories. (A) Network following HRS diet. Similarities (edges) within and between species, proteins, and metabolites (circles, squares, and triangles, respectively) across participants and time points, including only nodes significantly higher (red) or lower (blue) in HRS than baseline (two-sided *t* test; *P* < 0.05). (B) OPLS-DA plot of all data (features: metabolites, 5,552; proteome, 57,397; OTUs, 1,107) for baseline (blue, negative *x* axis) versus HRS (red, positive *x* axis); *P* = 8.3 × 10^−6^ (CV-ANOVA); *R*^2^*Y*(cum) = 0.96; *Q*^2^(cum) = 0.88. (C) OPLS-DA plot for HRS (red, negative *x* axis) versus LRS (green, positive *x* axis); *P* = 0.026 (CV-ANOVA); *R*^2^*Y*(cum) = 0.883; *Q*^2^(cum) = 0.534.

The true power of our study design comes from the ability to examine results across the different omics levels for an integrated systems picture. We used the context likelihood of relatedness (CLR) method (31) to display potential interactions of all three data sets, visualized as a network (Fig. 4A). Various areas of the network were assigned to different metabolite compound classes and related to taxa and proteins. We detected more discriminating features for the HRS diet compared to baseline (Fig. 4A) than for the LRS diet compared to baseline (Fig. S8), which again demonstrates that the HRS diet has a larger impact on the gut microbiome than the LRS diet. Some features were anticorrelated with the HRS diet, suggesting that they decreased with increased RS intake. These included 16S sequences corresponding to *Bacteroides* and *Lachnospiraceae* as well as some metabolites corresponding to unsaturated fatty acyls and some sterol lipids (Fig. 4A).

10.1128/mBio.01343-17.8 FIG S8  Integrative association network of the microbiome under low resistant starch diet. Download FIG S8, PDF file, 0.2 MB. Copyright © 2017 Maier et al. 2017 Maier et al. This content is distributed under the terms of the Creative Commons Attribution 4.0 International license.

The combined CLR data allowed us to identify features associated with a diet and features that were cocorrelated. This systems view of the metabolite composition and clustering confirms results from previous analyses of the influence of dietary resistant starch on some members of the *Firmicutes*, such as *F. prausnitzii* (32), and goes beyond them by also identifying correlations of specific species with specific metabolites and proteins. For example, not only was *F. prausnitzii* positively correlated with the HRS diet, but we found 14 novel polyketides and several unknown metabolites that were also correlated with this microorganism (Fig. 4A). Furthermore, *F. prausnitzii* was linked to a specific protein in the data set, phosphoenolpyruvate carboxykinase (ATP), which is an enzyme involved in several reactions of pyruvate metabolism. Changes in the abundance of *F. prausnitzii* have been linked to dysbiosis in several human disorders (33), and using our integrated systems approach, we are able to better understand its function *in vivo* by relating its changes in abundance to changes in protein and metabolite abundance (in both host and microbe) as a function of diet. Another example was *Ruminococcus* sp., which expressed an ABC sugar transporter (based on the proteome data) and was correlated with several unknown metabolites. We also found several additional specific metabolites that could be correlated with specific taxa (Table S1). To generalize this process of finding related features, we identified important modules of the network and listed their features in Table S2. Note that the majority of the metabolites remain unknown, illustrating the current challenge in identification of metabolites with various masses and isomers.

10.1128/mBio.01343-17.9 TABLE S1  OTUs with the most connections in the HRS network. Download TABLE S1, XLSX file, 0.01 MB. Copyright © 2017 Maier et al. 2017 Maier et al. This content is distributed under the terms of the Creative Commons Attribution 4.0 International license.

10.1128/mBio.01343-17.10 TABLE S2  Modules and features of the HRS network. Download TABLE S2, XLSX file, 0.03 MB. Copyright © 2017 Maier et al. 2017 Maier et al. This content is distributed under the terms of the Creative Commons Attribution 4.0 International license.

We complemented this network-based approach using a supervised ordination approach (Fig. 4B and C), combining the three data sets through a unit variance scaling. This method was able to discriminate metabolites, proteins, and OTUs that were correlated with each other and with the different diets. By examining the most abundant features for each data set (16S, proteome, and metabolome) we found numerous features that were cocorrelated with the HRS diet. For example, sterol lipids correlated with several *Ruminococcaceae*, *Clostridia*, and *Lachnospiraceae* species. On the other hand, numerous correlations of *Bacteroidetes*, *Lachnospiraceae*, and fatty acyls could be detected for the baseline diet. In general, in the HRS diet we see a highly significant increase in fatty acyls (HRS, 20.3%; LRS, 1.9%; baseline, 1.9%) and sterol lipids (HRS, 25.6%; LRS, 0.4%; baseline, 1.1%) and simultaneously an increase of *Faecalibacterium* (HRS, 1.4%; LRS and baseline, 0.0%) based on all significant features from the OPLS-DA results (Fig. 4B and C).

**FIG 5:**
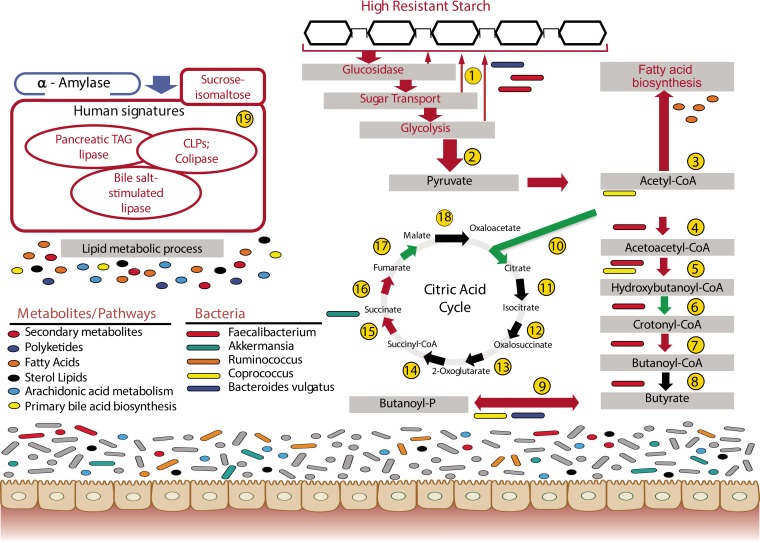
Overview of detected enzymes, pathways, species, and metabolites that were significantly impacted by a resistant starch diet. Red arrows/frames, increased in HRS; blue frames, decreased in HRS; black arrows, not detected or not increased in HRS over baseline; green arrows/frames, increased in LRS. 1, starch and sucrose metabolism; 2, glycolysis from glucose to pyruvate; 3, 3-oxoacyl-(acyl carrier protein) synthase; 4, acetyl-CoA acetyltransferase; 5, 3-hydroxyacyl-CoA dehydrogenase; 6, enoyl-CoA hydratase; 7, enoyl-(acyl carrier protein) reductase (NADH); 8, acetate CoA-transferase; 9, butyrate kinase; 10, citrate synthase; 11, aconitate hydratase; 12 and 13, isocitrate dehydrogenase; 14, 2-ketoglutarate ferredoxin oxidoreductase; 15, succinyl-CoA synthetase; 16, succinate dehydrogenase/fumarate reductase; 17, fumarate hydratase; 18, malate dehydrogenase; 19, human enzymes.

The multiomics data are summarized here in one overview model (Fig. 5) to illustrate the main effects of the resistant starch diet on the gut microbiome and functions that they carry out. Although some of the effects were previously predicted, such as changes in starch degradation and metabolism (1, 3), this is the first overview of the multitude of processes that occur using a systems approach to integrate different multiomics measurements simultaneously. For example, proteins involved in starch degradation and metabolism were increased in the HRS diet. Some enzymes, in particular human α-amylase, were significantly less abundant in the HRS diet. We hypothesize that this was due to the decrease in readily available starch compared to the baseline diet. This study also reinforced the importance of specific members of the *Firmicutes*, such as *F. prausnitzii*, for metabolism of nondietary carbohydrates in the diet, including enzymes for butyrate production by this organism. Some of the unexpected findings included links of *F. prausnitzii* to putative polyketide metabolites. In contrast, members of the *Bacteroidetes* were reduced in abundance following the HRS diet.

In summary, these results demonstrate that a multiomics approach provides a systems-level understanding of host and microbial metabolism and protein expression. These include novel links between an RS diet and lipid metabolism by the host and microbiome, beyond known impacts on short-chain fatty acid metabolism. The results also point toward key linkages between several members of the gut microbiome and metabolites and proteins produced in the gut. We present an overview of the multiomics data as a model of the complex interplay among organisms, metabolites, and functional processes. A notable strength of the approach used here is that proteins and metabolites were collected from host and microbiome simultaneously, allowing a systems-level approach to observing their interplay. Taken together, the results emphasize the importance of longitudinal, multiomics study designs for unraveling the effects of nutrition on the microbiome and health.

## MATERIALS AND METHODS

### Study design.

Twenty-six women and 13 men were enrolled in the present study. They were insulin resistant (HOMA-IR, >50th percentile for sex), had a body mass index (BMI) between 20 and 35 kg/m^2^ and met other eligibility criteria. Male participants (>20 years) and postmenopausal women (≥43 years, no menses for ≥3 years or no menses for ≥1 year and <3 years and additionally a follicle-stimulating hormone [FSH] plasma concentration within that range) were selected. Furthermore, all participants were nonsmokers, did not take any drugs (lipid- or glucose-lowering medications, blood-thinning agents, hormones, and antibiotics), had no record of cardiovascular disease (CVD) or other chronic diseases, and were otherwise healthy. Further clinical criteria are as follows: fasting glucose, <126 mg/dl; fasting triglycerides, <500 mg/dl; blood pressure, <150/90; low-density lipoprotein (LDL) and total cholesterol, ≤90th percentile for age and gender; 3 months prior to the study a stable weight with <3% change; and abstention from alcohol and any dietary supplements during the study. The study protocol was approved by the Institutional Review Board of Children’s Hospital and Research Center of Oakland. All participants gave written informed consent to take part in the study. The study protocols were approved by the Human Subjects Committee of both Children’s Hospital Oakland Research Institute and Lawrence Berkeley National Laboratory. The study design is presented in Fig. S1 in the supplemental material. Study participants were randomly assigned to either the high-carbohydrate (HC) or low-carbohydrate (LC) arm of the study and then assigned to a sequence of the two experimental diets that added resistant starch in proportion to carbohydrate load: for 2500 kcal/day menus, the low resistant starch diet contained 3 g in the LC arm and 4 g in the HC arm, while the high resistant starch (HRS) diet contained 48 g in the LC arm and 66 g in the HC arm. Each diet period was 2 weeks with a 2-week baseline washout diet in between. In both diets, type 2 resistant starch, a granular form of high-amylose cornstarch, was used. Melojel was used for LRS diets, while Hi-Maize 260 resistant starch was used for the HRS diets (National Starch). Fecal samples and fasting, postheparin, and postprandial blood samples were collected for the initial baseline diet and after each diet period.

### DNA extraction, library preparation, and sequencing.

DNA from the resulting 94 fecal samples (Fig. S1) was extracted in duplicate from 0.25-g samples using the PowerSoil DNA extraction kit (Mo Bio, Carlsbad, CA) according to the manufacturer’s instructions, plus an additional heat lysis step for 5 min at 60°C. The DNA was PCR amplified using the F515/R806 primer to target the V4-V6 region of the 16S rRNA gene and barcoded with a 12-base error-correcting Golay code as previously described by Caporaso et al. (34). Sequencing was performed on the Illumina HiSeq 2000 platform as previously described (35).

Sequence data were analyzed using the Quantitative Insights into Microbial Ecology (QIIME) pipeline. Briefly, sequences were quality filtered using default parameters and clustered into operational taxonomic units (OTUs) using the closed-reference OTU picking protocol at 97% sequencing identity (36). The taxonomy associated with each OTU was calculated as previously described (37).

The raw OTU table was filtered, normalized, and imported into R (38) using the phyloseq package (39). Samples were retained if they contained more than 5,000 reads. OTUs were retained if they appeared more than five times in more than 5 samples. These filtering techniques reduced the number of OTUs from 4,481 to 1,107 while preserving 98.6% of all observations. Using this filtered “biom” table, the DESeq2 package (40) was used to identify OTUs which were differentially abundant between groups. For significant OTUs at an alpha cutoff of 0.001 or 0.01, the log_2_ fold change was reported. To control for sequencing depth when calculating metrics of beta diversity and building the CLR interaction network, OTU counts in each sample were proportionally scaled to an even depth of 5,000 reads per sample.

### Combined protein and metabolite extraction.

Each stool sample (~15 g) was homogenized in a conical 50-ml Falcon tube with 20 ml cold sterile water using a handheld homogenizer (VDI 12 homogenizer, 115 V; VWR; catalog no. 82027-184) at full speed (30,000 rpm) for 2 periods of 30 s each, with cooling on ice between homogenization periods. The homogenate was proportioned into four 50-ml conical Falcon tubes per sample (2 tubes were processed for metabolomics and 2 for proteomics [see below]). For metabolite extraction, 2 portions were centrifuged at 4°C and 14,000 × *g* for 10 min, and the aqueous supernatant was decanted and stored at −80°C. For methanol extraction, 1.2 ml of cold (−20°C) methanol was added to each of the 2 cell pellets per sample and briefly mixed by vortexing. The cells were lysed by pressure cycling with 30 cycles at 30,000 lb/in^2^ using the Barocycler NEP3229 cell disruptor (Pressure Biosciences, Easton, MA). The lysates were centrifuged for 10 min at 14,000 × *g*, the two supernatants were combined into a fresh microcentrifuge tube, and the methanol extracts were stored at −80°C.

For protein extraction, 5 ml of PBS was added to the remaining two tubes of homogenized fecal material per sample, and the samples were briefly mixed by vortexing, followed by centrifugation at 4,000 × *g* for 5 min (4°C) to pellet larger debris. The supernatants were transferred to new 50-ml conical Falcon tubes on ice. An additional 4 ml of cold phosphate-buffered saline (PBS) was added to the cell pellet/debris per original tube and mixed with the homogenizer at full speed for 2 periods of 30 s each on ice. The combined supernatants were centrifuged for 10 min at 10,000 × *g* (4°C), and the supernatants were discarded. Each cell pellet was washed with cold PBS, resuspended in 600 µl of cold PBS, vortexed, and centrifuged at 14,000 × *g* for 10 min in a preweighed microcentrifuge tube. The supernatant was discarded, the pellet weight was calculated, and the sample was stored at −80°C.

### Metaproteomics approach.

The cell pellets were thawed and immediately diluted in 6 M guanidine-10 mM dithiothreitol (DTT), followed by heating at 60°C for 1 h with constant vortexing to dissolve the pellets. The samples were then diluted 6-fold with 50 mM Tris-10 mM CaCl_2_ (pH 7.6) and mixed by vortexing. Sequencing-grade trypsin (Promega, Madison, WI) was added to each sample at 1:100 (wt/wt) protein, and trypsin digestion was performed overnight at 37°C while gently mixing. An additional aliquot of trypsin was added to each sample at 1:100 (wt/wt) protein, and digestion was performed for an additional 4 h at 37°C. The digested samples were centrifuged for 15 min at 10,000 × *g* to remove particulate debris. Then, the samples were desalted using C_18_ Sep-Pak solid-phase extraction cartridges (Waters, Milford, MA) and concentrated to ~5 ml using a Savant SpeedVac (Thermo Fisher Scientific, Waltham, MA). The samples were then solvent exchanged with 0.1% formic acid in high-performance-liquid-chromatography (HPLC)-grade water and concentrated by vacuum to ~500 µl. The samples were filtered using Durapore polyvinylidene difluoride (PVDF) filters (0.45 µm; Millipore), aliquoted into 150-µl aliquots, and stored at −80°C prior to two-dimensional (2-D) liquid chromatography-tandem mass spectrometry (LC-MS/MS) analysis.

The resultant complex peptide mixtures (~150 µl) were loaded onto a biphasic C_18_-SCX (reverse-phase–strong cation exchange) (Phenomenex, Torrance, CA) self-packed nano-back column (3-cm C_18_, 3-cm SCX, 150-μm inside diameter [i.d.]) that serves as the first dimension of the 2-D LC system to capture peptides and wash away salts. Once loaded, the column was moved in line with a U3000 HPLC (Dionex, subsidiary of Thermo Scientific, Waltham, MA) which was split to obtain an ~300-nl/min flow rate over the nano-analytical columns. The back column was washed with 100% aqueous solvent followed by an organic solvent gradient (70% acetonitrile [ACN], 0.1% formic acid) to remove salts and move the peptides to the SCX phase. The back column was then attached to a 15-cm by 100-μm C_18_ front resolving column with an integrated nanospray tip (New Objective, Woburn, MA; Picofrit packed with Phenomenex Aqua C_18_). The resolving column was housed in a nanospray source (Proxeon; Thermo Fisher) attached to a QExactive mass spectrometer (Thermo Fisher, Bremen, Germany). An automated 24-h two-dimensional LC-MS/MS run was programmed into Xcalibur (Thermo Fisher), and each sample was analyzed with a separation scheme consisting of 12 salt pulses followed by 2-h C_18_ separation, as previously described (41). During each analysis and all sample runs, the QExactive settings were as follows: normalized collision energy for heated capillary dissociation (HCD) of 28 eV, a full-scan resolution of 70,000 from 400 to 1,600 *m/z*, an HCD MS/MS resolution of 17,500 with an isolation width of 3 *m/z*, and a dynamic exclusion setting of 15 s. Peptides were not excluded based on charge state, and 1 microscan for both full and MS/MS scans was acquired. All MS and MS/MS data were acquired in profile mode.

### Quantitation and normalization of metaproteome data.

All MS/MS spectra were searched against our customized sequence database (42), consisting of human protein sequences, translated metagenome sequences, proteins of 34 human-gut-isolated microbial species, and common contaminants (i.e., trypsin and keratin; 36 protein sequences). All MS/MS individual runs were searched with the SEQUEST (v.27) algorithm (43) against our customized FASTA sequence database, as previously described (42) (<4 miscleavages, 3-Da mass tolerance window around the precursor ion mass, and 0.5 Da for fragment ion masses). All SEQUEST output files were gathered and filtered using DTASelect (1.9) (44) at ≥2 peptides per protein and the following widely accepted parameters for all the MS runs: cross correlation (Xcorr) of at least 1.8, 2.5, and 3.5 for +1, +2, and +3 charge states, respectively, and a minimum delta normalized correlation (ΔCn) for 0.08. All the peptide spectrum matches (PSM) that could not satisfy a postdatabase search filter, ≥−10 to ≤10 ppm, were excluded to remove false positives as described previously (42). This resulted in a total of 57,397 proteins that were quantitatively identified (human, 1,103; microbes, 56,294). Spectrum counts (SC) of protein were normalized as described below (42):

$${\text{Normalized SC}}_{i}\;=\;\frac{\frac{\overset{M}{\underset{j\;=\;1}{\sum }}\overset{N}{\underset{k\;=\;1}{\sum }}{\text{SC}}_{k}}{M}}{\overset{N}{\underset{k\;=\;1}{\sum }}{\text{SC}}_{k}}\times \;{\text{SC}}_{i}$$

where *N* is the number of proteins, *M* is the number of MS runs, and *j*, *k*, and *i* are index values for each specific MS run, protein, and spectral count, respectively.

The metaproteome data were functionally analyzed by using Cluster of Orthologous Groups (COG) for microbial proteins and Kyoto Encyclopedia of Genes and Genomes (KEGG) pathway terms using COG software R 3.1.3 and Python 2.7.6.

### Metabolomics.

Methanol extracts of the stool samples were measured randomized in negative electrospray ionization mode [(–)ESI] using an ultrahigh-resolution SolariX Fourier transform ion cyclotron resonance mass spectrometer (FT-ICR-MS) (Bruker Daltonik GmbH) with a 12-tesla superconducting magnet and an Apollo II electrospray ionization (ESI) source. For each sample, 500 scans were acquired in single MS mode within a mass range from *m/z* 122.9 to *m/z* 1,000. The MS parameters were as follows: capillary, −3,600 V; nebulizer pressure, 200 kPa; dry gas, 4.0 liter/min; dry temperature, 180°C. The instrument was calibrated using a 5-ppm arginine solution.

#### (i) Metabolite data processing.

Ultrahigh-resolution mass spectra were processed using Data Analysis 4.0 SP2 (Bruker Daltonik GmbH). All spectra were calibrated internally using a reference list of known masses (fatty acids) with an error below 0.075 ppm and exported as ASCII files with a signal-to-noise ratio of 4 using Automation Engine 4.0 (Bruker Daltonik GmbH). ASCII files were converted to ASC files by in-house software, before all spectra were aligned to a data matrix with an error of 1 ppm by in-house software, resulting in 97,483 mass signals. The aligned data matrix was filtered by mass signals counted <5 times in *n* = 45 mass spectra and a mass defect above 0.8, which resulted in 14,167 mass signals. The mass signals were assigned to molecular formulas using NetCalc (27) (network tolerance, 0.2 ppm; NetCalc tolerance, 0.2 ppm) and searched against the KEGG (28) (Kyoto Encyclopedia of Genes and Genomes), HMDB (45) (Human Metabolome Database), and Lipid Maps (http://www.lipidmaps.org) databases using *Homo sapiens* as a reference organism using the MassTRIX web server (26, 46) with a maximum error of 1 ppm.

For multivariate data analysis (MVA), samples had been divided into 3 main groups (baseline [blue, G1], HRS [red, G2], and LRS [green, G3]), since orthogonal partial least-squares discriminant analysis (OPLS-DA) revealed no significant changes between the LRS diets at day 28 and day 56, as well as between the HRS diets at day 28 and day 56. Different OPLS-DA classification models were designed to evaluate the effect of resistant starch on the gut microbiome, which are listed in Table 1. The classification models were first validated by the 7-fold cross-validation method. In order to exclude overfitting, a cross-validation analysis of variance (CV-ANOVA) was applied for each OPLS-DA classification model. Further, the significance of each model (*P* value) and indicators such as the goodness-of-fit *R*^2^*Y*(cum) and the goodness-of-prediction *Q*^2^(cum) were subsequently reported. In order to evaluate the metabolomics data set with respect to the impact of the different diets on the human gut microbiome, all valid classification models of the OPLS-DA were merged to examine the most discriminating features among the baseline, HRS, and LRS diets.

In order to identify significant features of the metabolomics data set, a lipidomics-MS/MS approach was applied using an Acquity ultrahigh-performance liquid chromatography system (Waters GmbH, Eschborn, Germany) coupled to a Bruker maXis ultrahigh-resolution–time of flight mass spectrometer (UHR-TOF-MS) (Bruker Daltonik GmbH, Bremen, Germany) as previously described in the work of Witting et al. (47). Methanol (MeOH), acetonitrile (ACN), isopropanol (IPA), ammonium formate, and formic acid were of LC-MS quality and obtained from Sigma-Aldrich (Sigma-Aldrich GmbH, Taufkirchen, Germany). The water was purged through a Merck Millipore system with a resistance of 18 MΩ and a total organic carbon (TOC) of <4 ppb. Standards and a representative sample set were measured under the same conditions.

Mass spectra were processed and calibrated using Data Analysis 4.1 SR 1 (Bruker Daltonik GmbH). Chromatograms were averaged, made standard dependent, and calibrated using a reference list of standards of the injected calibration standard mix (G1969-85000; Agilent, Waldbronn, Germany), as well as the standards used for the MS/MS experiment with an error of less than 0.5 ppm. The extracted ion chromatograms (EIC) were extracted from each standard and representative sample with an error of ±0.01 Da.

For fatty acid identification, mass signals assigned as fatty acids were extracted from the data matrix and significantly changed fatty acids were visualized as box plots, displaying the intensity levels of each fatty acid between the baseline, HRS, and LRS diets, using RStudio (version 0.99.489). The significance was tested by applying the *post hoc* Kruskal-Nemenyi test for pairwise test of multiple comparisons of mean rank sums (PMCMR package, version 4.1) (48). Metabolites of the lipid metabolism altered by diet were visualized in a heat map by Hierarchical Clustering Explorer version 3.5 (49) (Human-Computer Interaction Lab, University of Maryland—College Park). Therefore, the data were normalized (*X* − *m*/σ) and clustered by rows with Euclidean distance.

#### (ii) Short-chain fatty acid analysis.

The fecal MeOH extracts and chemical standards of propionic acid, butyric acid, valeric acid, and isovaleric acid were prepared and derivatized as instructed in the AMP^+^ mass spectrometry kit (Cayman Chemicals) product insert. Each mixture was diluted with 352 µl A-B (5 mM CH_3_COONH_4_ plus 0.1% acetic acid-ACN in a 99:1 ratio.

SCFA analysis was performed on an Acquity ultrahigh-performance liquid chromatography system (Waters GmbH, Eschborn, Germany) coupled to a Bruker maXis UHR-TOF-MS (Bruker Daltonik GmbH, Bremen, Germany), and SCFA were measured in positive electrospray ionization mode. Gradient separation of 1 µl took place on a Waters BEH C_8_ column (1.7 µm, 2.1 mm by 150 mm) with A (5 mM CH_3_COONH_4_ plus 0.1% acetic acid) and B (100% ACN). Total run time was 22 min plus 2 min prerun. Start conditions of the gradient separation were 99% A. This was held for 1 min and then decreased to 1% A within 16 min and held for 2 min. A was increased to 99% A for 0.2 min and held for 2.8 min. The flow rate was 0.3 ml/min, and column temperature was 40°C. MS parameters were as follows: mass range, *m/z* 50 to 1,200; nebulizer gas, 200 kPa; dry gas, 8 liters/min; dry temperature, 200°C; spectrum rate, 2.0 Hz; capillary, 4,500 V; end plate offset, −500 V. Simultaneously, a photodiode array detector (PDA) was operated at a UV range from 190 to 500 nm. For calibration, a 1:4-diluted ESI-L low-concentration tuning mix (Agilent, Waldbronn, Germany) was injected prior to the separation at the first 0.1 min of the analysis.

The adducts of the derivatized products were calculated as follows: *M* (monoisotopic mass [metabolite]) − H_2_O + AMP^+^ (C_12_H_13_N_2_^+^) = *M* − AMP^+^. The retention time (RT) was extracted by Data Analysis version 4.1 (Bruker Daltonik GmbH, Bremen, Germany), and the peak areas were extracted by QuantAnalysis version 2.1 (Bruker Daltonik GmbH, Bremen, Germany). SCFA were quantified by external calibration including 8 calibration points based on the extracted peak areas of each standard concentration via the calculated calibration function (Table 2).

**TABLE 2:** External calibration results of the SCFA analysis

Name	*m/z* (derivatized)	RT (min)	Calibration function	Coefficient of determination (*R*^2^)	Method
Propionic acid	2,411,341	4.0	*y* = 1.2119*x* − 0.5784	0.9990	UV
Butyric acid	2,551,497	4.6	*y* = 59,940*x* + 16,956	0.9981	MS
Isovaleric acid	2,691,654	5.3	*y* = 82,730*x* + 6,202.9	0.9993	MS
Valeric acid	2,691,654	5.5	*y* = 92,248*x* + 4,883	0.9998	MS

### Visualization of microbiome and proteome data.

The microbiome and proteome data were visually presented based on Voronoi treemaps, developed and adapted for biological applications at the Greifswald University Institute for Microbiology (50–52). The treemaps originate from the work of Ben Shneiderman (University of Michigan) (53), followed by an improvement to Voronoi treemaps performed by Balzer and Deussen (54), and were adapted for applications in biosciences (50).

The protein data were condensed to the microbial species level, and the Voronoi treemaps were colored accordingly to species (55). In order to assign the proteins to functional classes, all proteins were analyzed separately (microbial to COG and human to KEGG Brite [56, 57]). Pearson correlation coefficients were calculated based on RS amounts versus relative abundances of OTUs or proteins in the samples. For these calculations, values approximating the concentrations of RS in the diets were assigned as follows: 0 for baseline, 0.05 (3 g RS) for LRS, and 1 (48 g RS) for HRS. For the rarefied OTU data, the correlation coefficients were averaged and visualized using the same color code as applied to the proteome data, and the treemap polygon sizes correspond to the average counts of OTUs for all samples to visualize their relative amounts in the entire data set.

### Multiomics integrative analyses.

Pairs of data sets were assembled by matching participants from each individual data set (16S, proteomics, and metabolomics) to provide maximum overlapping data sets (16S plus proteomics, proteomics plus metabolomics, and metabolomics plus 16S). All individual and combined data sets were then filtered to exclude those rows (OTU, protein, or metabolite, respectively) that had greater than 50% of values missing. The context likelihood of relatedness (CLR) method was applied to determine shared information for all pairs of rows (31, 58). Six individual networks were constructed by applying a *Z* score filter of 6.5 to each comparison: protein plus protein, 16S plus 16S, metabolomics plus metabolomics, 16S plus protein, protein plus metabolomics, and 16S plus metabolomics. Edges from individual networks were combined into a single network taking interactions from within a data set (e.g., protein to protein) from the networks inferred from single data sets (e.g., proteomics) and inter-data-set edges from the appropriate combined data sets (protein to metabolite edges from the proteomics-plus-metabolomics data set). Networks were represented in Cytoscape (59), and annotations from the individual data types were used to highlight clusters of components enriched in particular labels as indicated in the figures.

Multiomics integration was done using SIMCA-P 13.0.3.0 (Umetrics, Umeå, Sweden). In order to study the three combined data sets, two different OPLS-DA models were built: the baseline diet to the HRS diet and the HRS diet to the LRS diet. For integration of all the different omics data sets, the samples were aligned in one matrix and were unit variance (UV) scaled. OPLS-DA loading plots were constructed to simultaneously visualize features of the genome, proteome, and metabolome impacted by baseline, LRS, or HRS diet. The loadings were extracted and visualized as loading plots using RStudio (version 0.99.489) (60).

### Data availability.

Sequencing data are available on Qiita (https://qiita.ucsd.edu/study/description/1191) and the EBI-ENA accession is ERP104494. Proteomics analysis data are available on Zenodo (https://zenodo.org/record/838741). 

## References

[1] Scott KP, Gratz SW, Sheridan PO, Flint HJ, Duncan SH 2013 The influence of diet on the gut microbiota. Pharmacol Res 69:52–60. doi:10.1016/j.phrs.2012.10.020. 23147033

[2] Walker AW, Ince J, Duncan SH, Webster LM, Holtrop G, Ze X, Brown D, Stares MD, Scott P, Bergerat A, Louis P, McIntosh F, Johnstone AM, Lobley GE, Parkhill J, Flint HJ 2011 Dominant and diet-responsive groups of bacteria within the human colonic microbiota. ISME J 5:220–230. doi:10.1038/ismej.2010.118. 20686513 PMC3105703

[3] Flint HJ, Scott KP, Duncan SH, Louis P, Forano E 2012 Microbial degradation of complex carbohydrates in the gut. Gut Microbes 3:289–306. doi:10.4161/gmic.19897. 22572875 PMC3463488

[4] Upadhyaya B, McCormack L, Fardin-Kia AR, Juenemann R, Nichenametla S, Clapper J, Specker B, Dey M 2016 Impact of dietary resistant starch type 4 on human gut microbiota and immunometabolic functions. Sci Rep 6:28797. doi:10.1038/srep28797. 27356770 PMC4928084

[5] Martínez I, Kim J, Duffy PR, Schlegel VL, Walter J 2010 Resistant starches types 2 and 4 have differential effects on the composition of the fecal microbiota in human subjects. PLoS One 5:e15046. doi:10.1371/journal.pone.0015046. 21151493 PMC2993935

[6] Gao Z, Yin J, Zhang J, Ward RE, Martin RJ, Lefevre M, Cefalu WT, Ye J 2009 Butyrate improves insulin sensitivity and increases energy expenditure in mice. Diabetes 58:1509–1517. doi:10.2337/db08-1637. 19366864 PMC2699871

[7] Louis P, Flint HJ 2009 Diversity, metabolism and microbial ecology of butyrate-producing bacteria from the human large intestine. FEMS Microbiol Lett 294:1–8. doi:10.1111/j.1574-6968.2009.01514.x. 19222573

[8] Sokol H, Pigneur B, Watterlot L, Lakhdari O, Bermúdez-Humarán LG, Gratadoux JJ, Blugeon S, Bridonneau C, Furet JP, Corthier G, Grangette C, Vasquez N, Pochart P, Trugnan G, Thomas G, Blottière HM, Doré J, Marteau P, Seksik P, Langella P 2008 Faecalibacterium prausnitzii is an anti-inflammatory commensal bacterium identified by gut microbiota analysis of Crohn disease patients. Proc Natl Acad Sci U S A 105:16731–16736. doi:10.1073/pnas.0804812105. 18936492 PMC2575488

[9] Ze X, Duncan SH, Louis P, Flint HJ 2012 Ruminococcus bromii is a keystone species for the degradation of resistant starch in the human colon. ISME J 6:1535–1543. doi:10.1038/ismej.2012.4. 22343308 PMC3400402

[10] Khan MT, Duncan SH, Stams AJ, van Dijl JM, Flint HJ, Harmsen HJ 2012 The gut anaerobe Faecalibacterium prausnitzii uses an extracellular electron shuttle to grow at oxic-anoxic interphases. ISME J 6:1578–1585. doi:10.1038/ismej.2012.5. 22357539 PMC3400418

[11] Cockburn DW, Orlovsky NI, Foley MH, Kwiatkowski KJ, Bahr CM, Maynard M, Demeler B, Koropatkin NM 2015 Molecular details of a starch utilization pathway in the human gut symbiont Eubacterium rectale. Mol Microbiol 95:209–230. doi:10.1111/mmi.12859. 25388295 PMC4437465

[12] Verberkmoes NC, Russell AL, Shah M, Godzik A, Rosenquist M, Halfvarson J, Lefsrud MG, Apajalahti J, Tysk C, Hettich RL, Jansson JK 2009 Shotgun metaproteomics of the human distal gut microbiota. ISME J 3:179–189. doi:10.1038/ismej.2008.108. 18971961

[13] Jansson J, Willing B, Lucio M, Fekete A, Dicksved J, Halfvarson J, Tysk C, Schmitt-Kopplin P 2009 Metabolomics reveals metabolic biomarkers of Crohn’s disease. PLoS One 4:e6386. doi:10.1371/journal.pone.0006386. 19636438 PMC2713417

[14] Chiu S, Williams PT, Dawson T, Bergman RN, Stefanovski D, Watkins SM, Krauss RM 2014 Diets high in protein or saturated fat do not affect insulin sensitivity or plasma concentrations of lipids and lipoproteins in overweight and obese adults. J Nutr 144:1753–1759. doi:10.3945/jn.114.197624. 25332473 PMC4195419

[15] Raben A, Tagliabue A, Christensen NJ, Madsen J, Holst JJ, Astrup A 1994 Resistant starch: the effect on postprandial glycemia, hormonal response, and satiety. Am J Clin Nutr 60:544–551. 8092089 10.1093/ajcn/60.4.544

[16] Sands AL, Leidy HJ, Hamaker BR, Maguire P, Campbell WW 2009 Consumption of the slow-digesting waxy maize starch leads to blunted plasma glucose and insulin response but does not influence energy expenditure or appetite in humans. Nutr Res 29:383–390. doi:10.1016/j.nutres.2009.05.009. 19628104 PMC4562027

[17] Behall KM, Hallfrisch J 2002 Plasma glucose and insulin reduction after consumption of breads varying in amylose content. Eur J Clin Nutr 56:913–920. doi:10.1038/sj.ejcn.1601411. 12209381

[18] Robertson MD, Bickerton AS, Dennis AL, Vidal H, Frayn KN 2005 Insulin-sensitizing effects of dietary resistant starch and effects on skeletal muscle and adipose tissue metabolism. Am J Clin Nutr 82:559–567. 16155268 10.1093/ajcn.82.3.559

[19] Bergeron N, Williams PT, Lamendella R, Faghihnia N, Grube A, Li X, Wang Z, Knight R, Jansson JK, Hazen SL, Krauss RM 2016 Diets high in resistant starch increase plasma levels of trimethylamine-*N*-oxide, a gut microbiome metabolite associated with CVD risk. Br J Nutr 116:2020–2029. doi:10.1017/S0007114516004165. 27993177 PMC5885763

[20] Miquel S, Leclerc M, Martin R, Chain F, Lenoir M, Raguideau S, Hudault S, Bridonneau C, Northen T, Bowen B, Bermúdez-Humarán LG, Sokol H, Thomas M, Langella P 2015 Identification of metabolic signatures linked to anti-inflammatory effects of Faecalibacterium prausnitzii. mBio 6:e00300-15. doi:10.1128/mBio.00300-15. 25900655 PMC4453580

[21] Louis P, Young P, Holtrop G, Flint HJ 2010 Diversity of human colonic butyrate-producing bacteria revealed by analysis of the butyryl-CoA:acetate CoA-transferase gene. Environ Microbiol 12:304–314. doi:10.1111/j.1462-2920.2009.02066.x. 19807780

[22] Qin J, Li Y, Cai Z, Li S, Zhu J, Zhang F, Liang S, Zhang W, Guan Y, Shen D, Peng Y, Zhang D, Jie Z, Wu W, Qin Y, Xue W, Li J, Han L, Lu D, Wu P, Dai Y, Sun X, Li Z, Tang A, Zhong S, Li X, Chen W, Xu R, Wang M, Feng Q, Gong M, Yu J, Zhang Y, Zhang M, Hansen T, Sanchez G, Raes J, Falony G, Okuda S, Almeida M, LeChatelier E, Renault P, Pons N, Batto JM, Zhang Z, Chen H, Yang R, Zheng W, Li S, Yang H 2012 A metagenome-wide association study of gut microbiota in type 2 diabetes. Nature 490:55–60. doi:10.1038/nature11450. 23023125

[23] Belenguer A, Duncan SH, Calder AG, Holtrop G, Louis P, Lobley GE, Flint HJ 2006 Two routes of metabolic cross-feeding between Bifidobacterium adolescentis and butyrate-producing anaerobes from the human gut. Appl Environ Microbiol 72:3593–3599. doi:10.1128/AEM.72.5.3593-3599.2006. 16672507 PMC1472403

[24] Rendleman JA Jr. 2000 Hydrolytic action of alpha-amylase on high-amylose starch of low molecular mass. Biotechnol Appl Biochem 31:171–178. 10814586 10.1042/ba19990100

[25] Ramsay AG, Scott KP, Martin JC, Rincon MT, Flint HJ 2006 Cell-associated alpha-amylases of butyrate-producing firmicute bacteria from the human colon. Microbiology 152:3281–3290. doi:10.1099/mic.0.29233-0. 17074899

[26] Suhre K, Schmitt-Kopplin P 2008 MassTRIX: mass translator into pathways. Nucleic Acids Res 36:W481–W484. doi:10.1093/nar/gkn194. 18442993 PMC2447776

[27] Tziotis D, Hertkorn N, Schmitt-Kopplin P 2011 Kendrick-analogous network visualisation of ion cyclotron resonance Fourier transform mass spectra: improved options for the assignment of elemental compositions and the classification of organic molecular complexity. Eur J Mass Spectrom 17:415–421. doi:10.1255/ejms.1135. 22006638

[28] Kanehisa M, Goto S 2000 KEGG: Kyoto encyclopedia of genes and genomes. Nucleic Acids Res 28:27–30. doi:10.1093/nar/28.1.27. 10592173 PMC102409

[29] Bowen BP, Northen TR 2010 Dealing with the unknown: metabolomics and metabolite atlases. J Am Soc Mass Spectrom 21:1471–1476. doi:10.1016/j.jasms.2010.04.003. 20452782

[30] Scalbert A, Brennan L, Fiehn O, Hankemeier T, Kristal BS, van Ommen B, Pujos-Guillot E, Verheij E, Wishart D, Wopereis S 2009 Mass-spectrometry-based metabolomics: limitations and recommendations for future progress with particular focus on nutrition research. Metabolomics 5:435–458. doi:10.1007/s11306-009-0168-0. 20046865 PMC2794347

[31] Faith JJ, Hayete B, Thaden JT, Mogno I, Wierzbowski J, Cottarel G, Kasif S, Collins JJ, Gardner TS 2007 Large-scale mapping and validation of Escherichia coli transcriptional regulation from a compendium of expression profiles. PLoS Biol 5:e8. doi:10.1371/journal.pbio.0050008. 17214507 PMC1764438

[32] Haenen D, Zhang J, Souza da Silva C, Bosch G, van der Meer IM, van Arkel J, van den Borne JJ, Pérez Gutiérrez O, Smidt H, Kemp B, Müller M, Hooiveld GJ 2013 A diet high in resistant starch modulates microbiota composition, SCFA concentrations, and gene expression in pig intestine. J Nutr 143:274–283. doi:10.3945/jn.112.169672. 23325922

[33] Miquel S, Martín R, Rossi O, Bermúdez-Humarán LG, Chatel JM, Sokol H, Thomas M, Wells JM, Langella P 2013 Faecalibacterium prausnitzii and human intestinal health. Curr Opin Microbiol 16:255–261. doi:10.1016/j.mib.2013.06.003. 23831042

[34] Caporaso JG, Lauber CL, Walters WA, Berg-Lyons D, Lozupone CA, Turnbaugh PJ, Fierer N, Knight R 2011 Global patterns of 16S rRNA diversity at a depth of millions of sequences per sample. Proc Natl Acad Sci U S A 108:4516–4522. doi:10.1073/pnas.1000080107. 20534432 PMC3063599

[35] Caporaso JG, Lauber CL, Walters WA, Berg-Lyons D, Huntley J, Fierer N, Owens SM, Betley J, Fraser L, Bauer M, Gormley N, Gilbert JA, Smith G, Knight R 2012 Ultra-high-throughput microbial community analysis on the Illumina HiSeq and MiSeq platforms. ISME J 6:1621–1624. doi:10.1038/ismej.2012.8. 22402401 PMC3400413

[36] Caporaso JG, Kuczynski J, Stombaugh J, Bittinger K, Bushman FD, Costello EK, Fierer N, Peña AG, Goodrich JK, Gordon JI, Huttley GA, Kelley ST, Knights D, Koenig JE, Ley RE, Lozupone CA, McDonald D, Muegge BD, Pirrung M, Reeder J, Sevinsky JR, Turnbaugh PJ, Walters WA, Widmann J, Yatsunenko T, Zaneveld J, Knight R 2010 QIIME allows analysis of high-throughput community sequencing data. Nat Methods 7:335–336. doi:10.1038/nmeth.f.303. 20383131 PMC3156573

[37] Lozupone C, Knight R 2005 UniFrac: a new phylogenetic method for comparing microbial communities. Appl Environ Microbiol 71:8228–8235. doi:10.1128/AEM.71.12.8228-8235.2005. 16332807 PMC1317376

[38] R Foundation for Statistical Computing 2014 R: a language and environment for statistical computing. R Foundation for Statistical Computing, Vienna, Austria http://www.R-project.org/.

[39] McMurdie PJ, Holmes S 2013 phyloseq: an R package for reproducible interactive analysis and graphics of microbiome census data. PLoS One 8:e61217. doi:10.1371/journal.pone.0061217. 23630581 PMC3632530

[40] Love MI, Huber W, Anders S 2014 Moderated estimation of fold change and dispersion for RNA-seq data with DESeq2. Genome Biol 15:550. doi:10.1186/s13059-014-0550-8. 25516281 PMC4302049

[41] Schleicher TR, VerBerkmoes NC, Shah M, Nyholm SV 2014 Colonization state influences the hemocyte proteome in a beneficial squid-Vibrio symbiosis. Mol Cell Proteomics 13:2673–2686. doi:10.1074/mcp.M113.037259. 25038065 PMC4188995

[42] Erickson AR, Cantarel BL, Lamendella R, Darzi Y, Mongodin EF, Pan C, Shah M, Halfvarson J, Tysk C, Henrissat B, Raes J, Verberkmoes NC, Fraser CM, Hettich RL, Jansson JK 2012 Integrated metagenomics/metaproteomics reveals human host-microbiota signatures of Crohn’s disease. PLoS One 7:e49138. doi:10.1371/journal.pone.0049138. 23209564 PMC3509130

[43] Eng JK, McCormack AL, Yates JR 1994 An approach to correlate tandem mass spectral data of peptides with amino acid sequences in a protein database. J Am Soc Mass Spectrom 5:976–989. doi:10.1016/1044-0305(94)80016-2. 24226387

[44] Tabb DL, McDonald WH, Yates JR III 2002 DTASelect and Contrast: tools for assembling and comparing protein identifications from shotgun proteomics. J Proteome Res 1:21–26. doi:10.1021/pr015504q. 12643522 PMC2811961

[45] Wishart DS, Tzur D, Knox C, Eisner R, Guo AC, Young N, Cheng D, Jewell K, Arndt D, Sawhney S, Fung C, Nikolai L, Lewis M, Coutouly MA, Forsythe I, Tang P, Shrivastava S, Jeroncic K, Stothard P, Amegbey G, Block D, Hau DD, Wagner J, Miniaci J, Clements M, Gebremedhin M, Guo N, Zhang Y, Duggan GE, Macinnis GD, Weljie AM, Dowlatabadi R, Bamforth F, Clive D, Greiner R, Li L, Marrie T, Sykes BD, Vogel HJ, Querengesser L 2007 HMDB: the human metabolome database. Nucleic Acids Res 35:D521–D526. doi:10.1093/nar/gkl923. 17202168 PMC1899095

[46] Wägele B, Witting M, Schmitt-Kopplin P, Suhre K 2012 MassTRIX reloaded: combined analysis and visualization of transcriptome and metabolome data. PLoS One 7:e39860. doi:10.1371/journal.pone.0039860. 22815716 PMC3391204

[47] Witting M, Maier TV, Garvis S, Schmitt-Kopplin P 2014 Optimizing a ultrahigh pressure liquid chromatography-time of flight-mass spectrometry approach using a novel sub-2mµm core-shell particle for in depth lipidomic profiling of Caenorhabditis elegans. J Chromatogr A 1359:91–99. doi:10.1016/j.chroma.2014.07.021. 25074420

[48] Pohlert T 2014 The pairwise Multiple Comparison of Mean Ranks Package (PMCMR), R package, version 4.1. https://CRAN.R-project.org/package=PMCMR.

[49] Seo J, Shneiderman B 2002 Interactively exploring hierarchical clustering results. Computer 35:80.

[50] Bernhardt J, Funke S, Hecker M, Siebourg J 2009 Visualizing gene expression data via Voronoi treemaps, p 233–241. *In* ISVD ’09. Sixth Int Symp Voronoi Diagrams. IEEE, New York, NY http://doi.ieeecomputersociety.org/10.1109/ISVD.2009.33.

[51] Mehlan H, Schmidt F, Weiss S, Schüler J, Fuchs S, Riedel K, Bernhardt J 2013 Data visualization in environmental proteomics. Proteomics 13:2805–2821. doi:10.1002/pmic.201300167. 23913834

[52] Otto A, Bernhardt J, Meyer H, Schaffer M, Herbst FA, Siebourg J, Mäder U, Lalk M, Hecker M, Becher D 2010 Systems-wide temporal proteomic profiling in glucose-starved Bacillus subtilis. Nat Commun 1:137. doi:10.1038/ncomms1137. 21266987 PMC3105300

[53] Shneiderman B 1992 Tree visualization with tree-maps: 2-d space-filling approach. ACM Trans Graph 11:92–99. doi:10.1145/102377.115768.

[54] Balzer M, Deussen O 2005 Voronoi treemaps, p 49–56. *In* InfoVis 2005. IEEE Symp Inf Vis. IEEE, New York, NY. doi:10.1109/INFVIS.2005.1532128.

[55] Human Microbiome Project Consortium 2012 Structure, function and diversity of the healthy human microbiome. Nature 486:207–214. doi:10.1038/nature11234. 22699609 PMC3564958

[56] Kanehisa M, Goto S, Sato Y, Furumichi M, Tanabe M 2012 KEGG for integration and interpretation of large-scale molecular data sets. Nucleic Acids Res 40:D109–D114. doi:10.1093/nar/gkr988. 22080510 PMC3245020

[57] Kanehisa M, Furumichi M, Tanabe M, Sato Y, Morishima K 2017 KEGG: new perspectives on genomes, pathways, diseases and drugs. Nucleic Acids Res 45:D353–D361. doi:10.1093/nar/gkw1092. 27899662 PMC5210567

[58] McDermott JE, Vartanian KB, Mitchell H, Stevens SL, Sanfilippo A, Stenzel-Poore MP 2012 Identification and validation of Ifit1 as an important innate immune bottleneck. PLoS One 7:e36465. doi:10.1371/journal.pone.0036465. 22745654 PMC3380000

[59] Shannon P, Markiel A, Ozier O, Baliga NS, Wang JT, Ramage D, Amin N, Schwikowski B, Ideker T 2003 Cytoscape: a software environment for integrated models of biomolecular interaction networks. Genome Res 13:2498–2504. doi:10.1101/gr.1239303. 14597658 PMC403769

[60] R Team 2015 RStudio: integrated development for R, v0.99.489. RStudio, Inc, Boston, MA http://www.rstudio.com/.

