# Supplementary figures and images for: Impact of Dietary Resistant Starch on the Human Gut Microbiome, Metaproteome, and Metabolome
Source: mBio. 2017 Oct 17;8(5):e01343-17. doi: 10.1128/mBio.01343-17 (PMC5646248; doi:10.1128/mBio.01343-17)

A

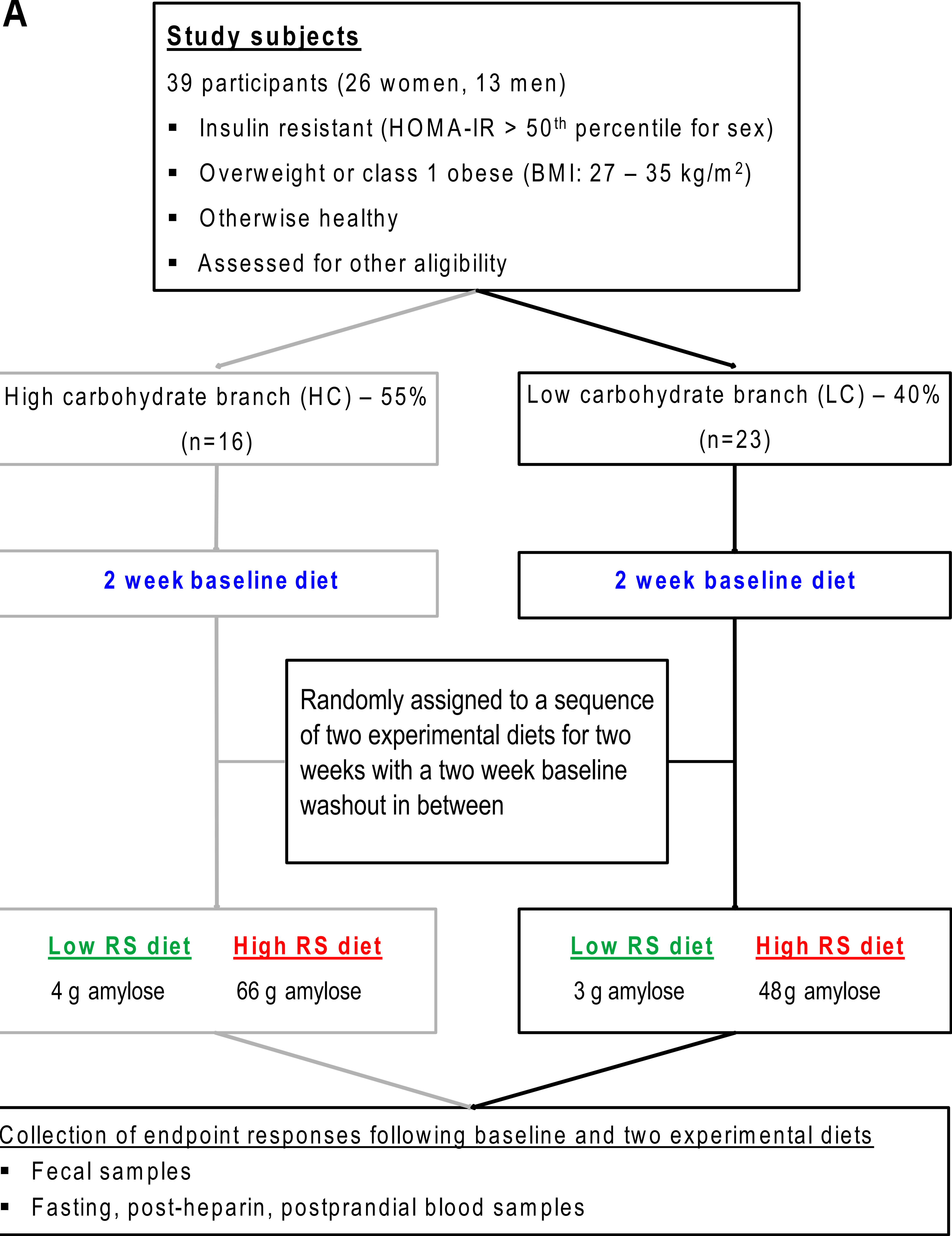

B

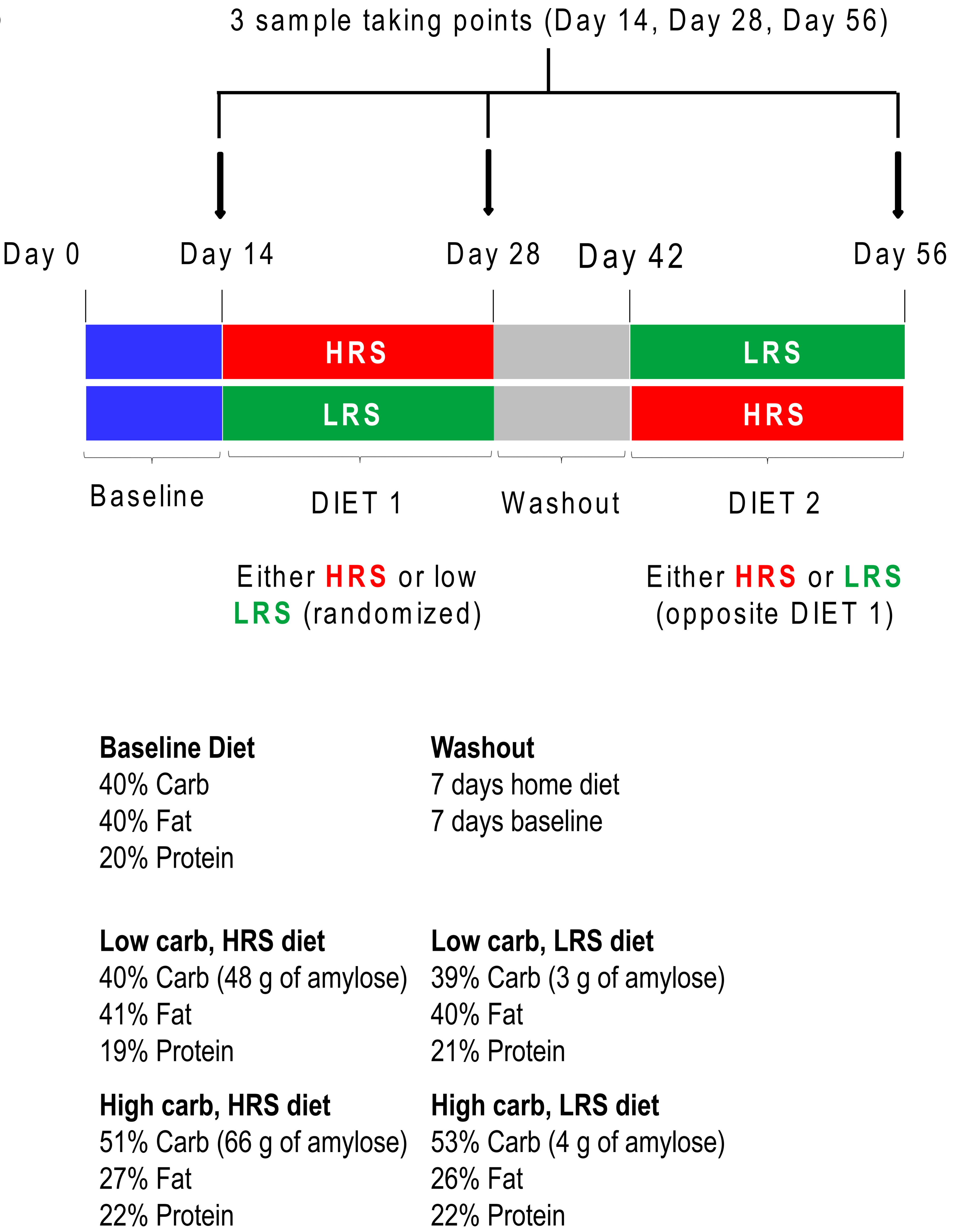

Supplement: FIG S1 [file mbo001173530sf1.pdf]

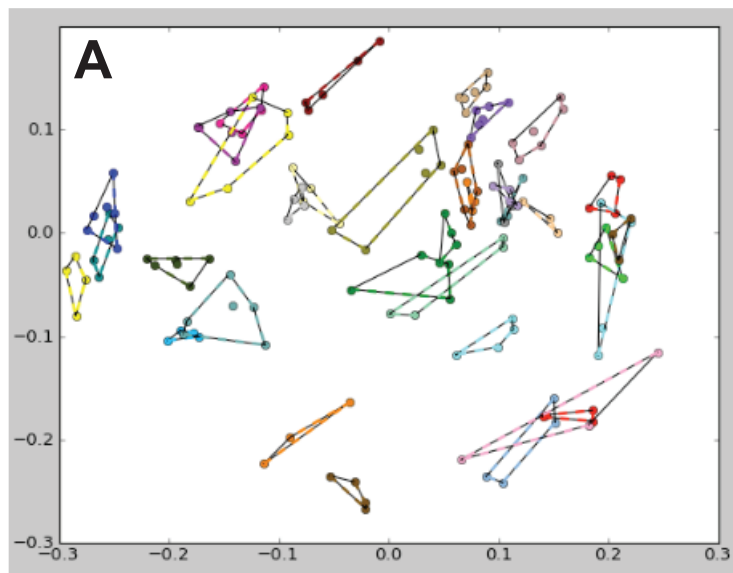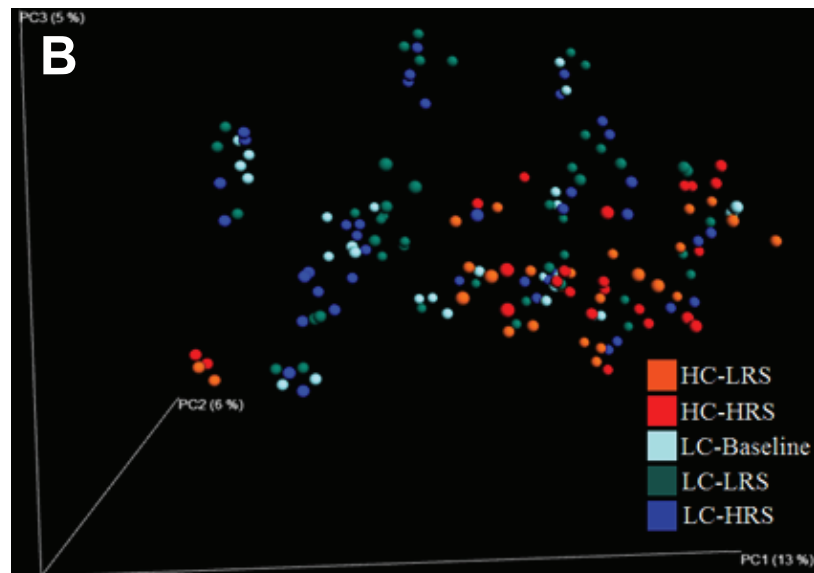

Supplement: FIG S2 [file mbo001173530sf2.pdf]

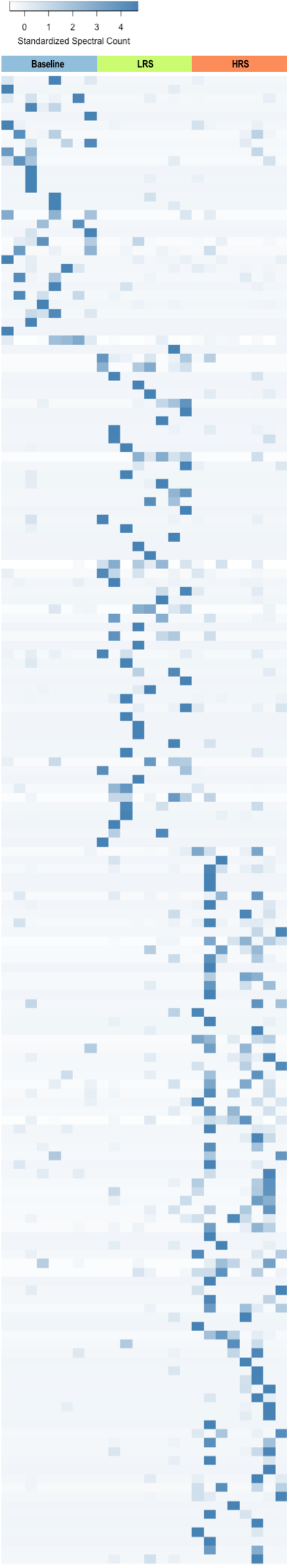

Supplement: FIG S4 [file mbo001173530sf4.pdf]

**A**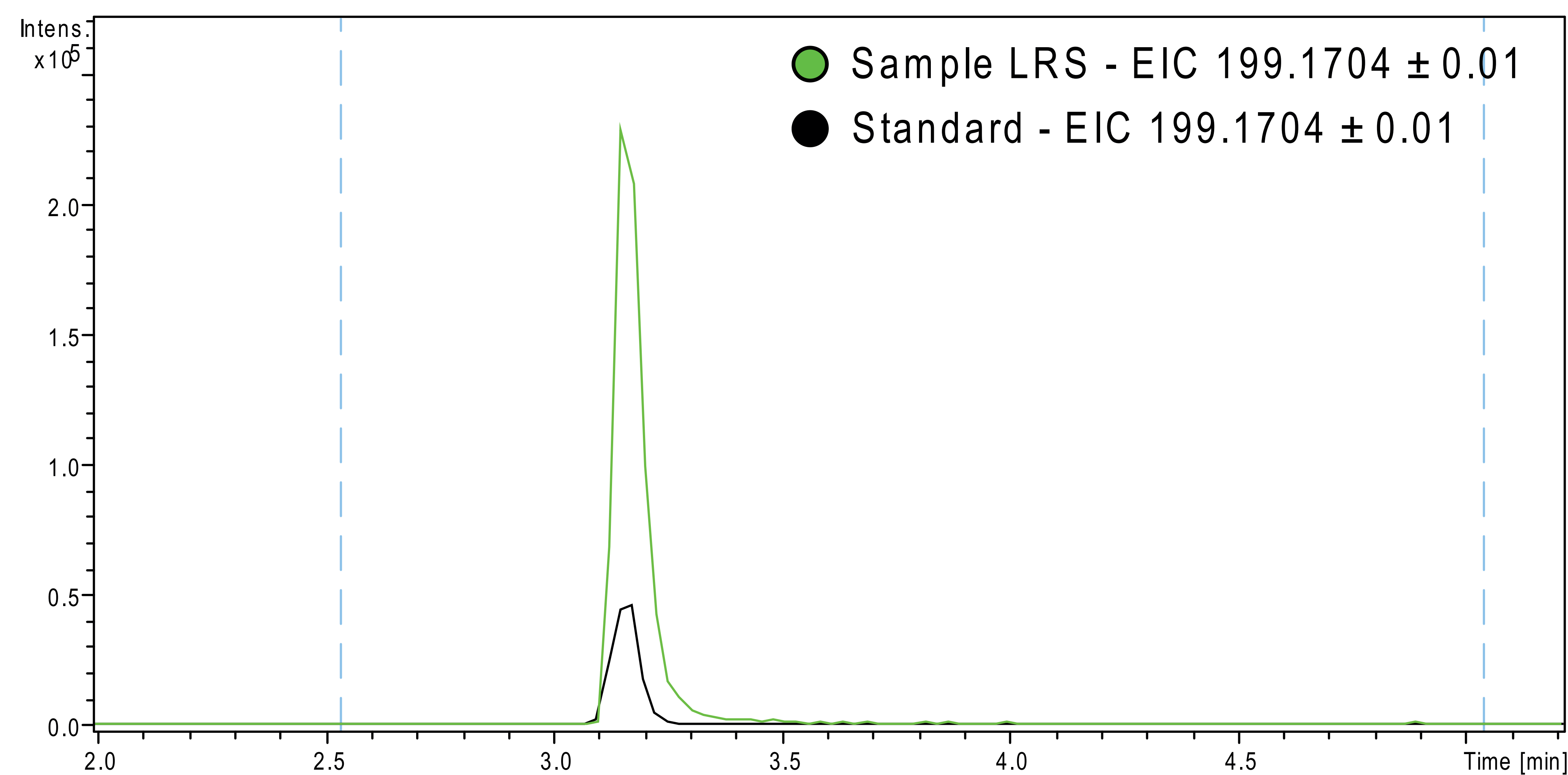**UPLC-QToF-MS**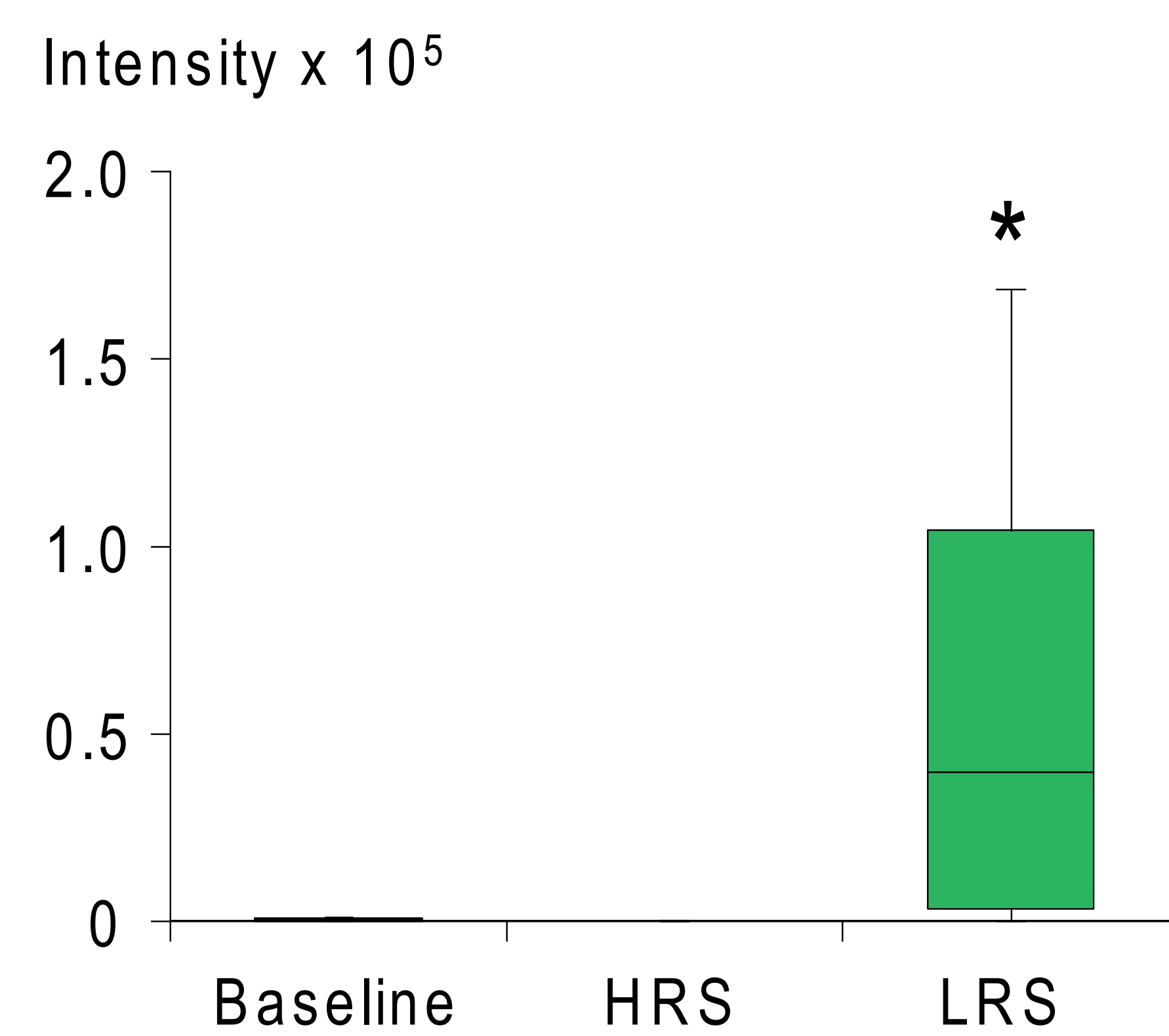**FT-ICR-MS**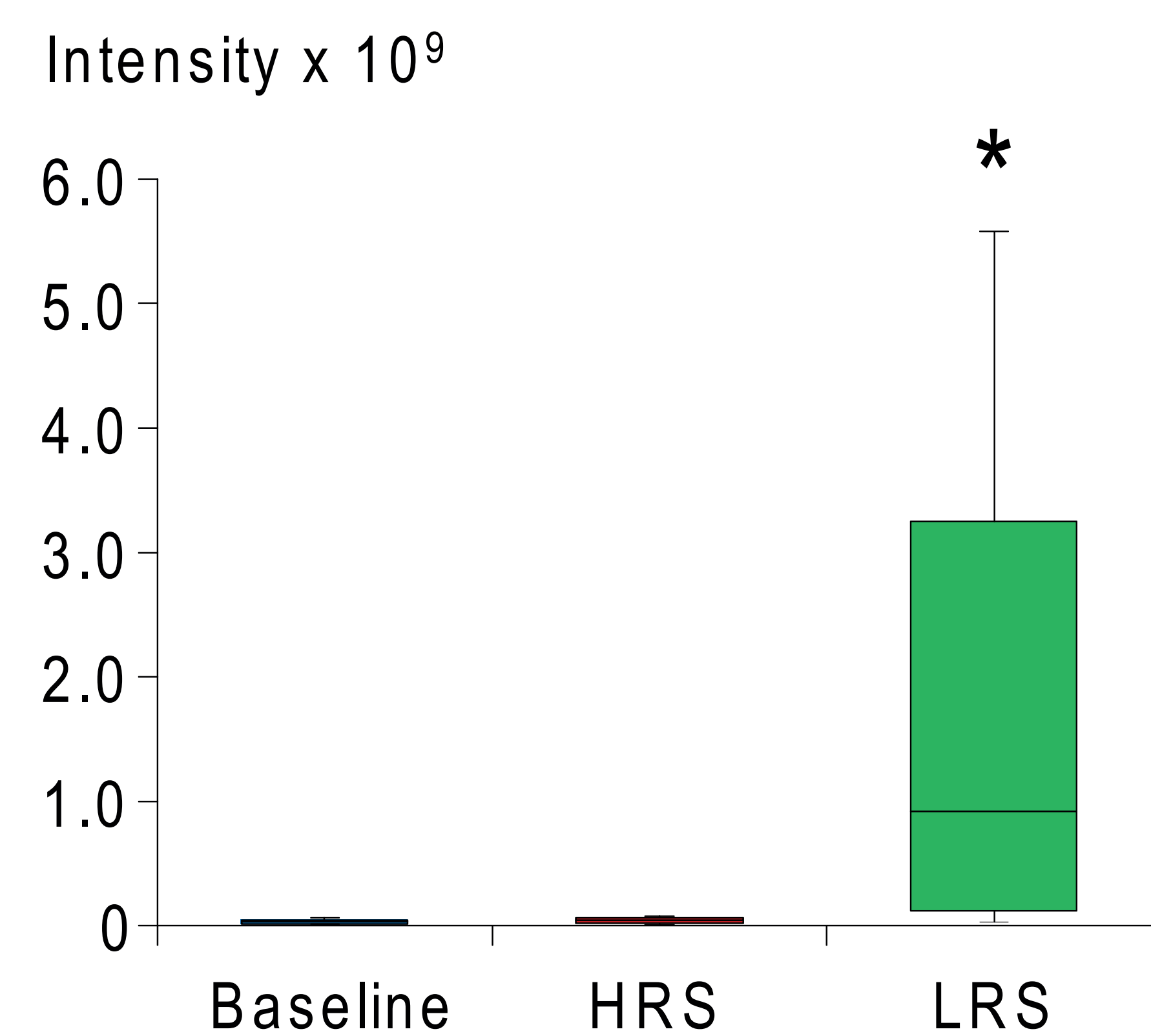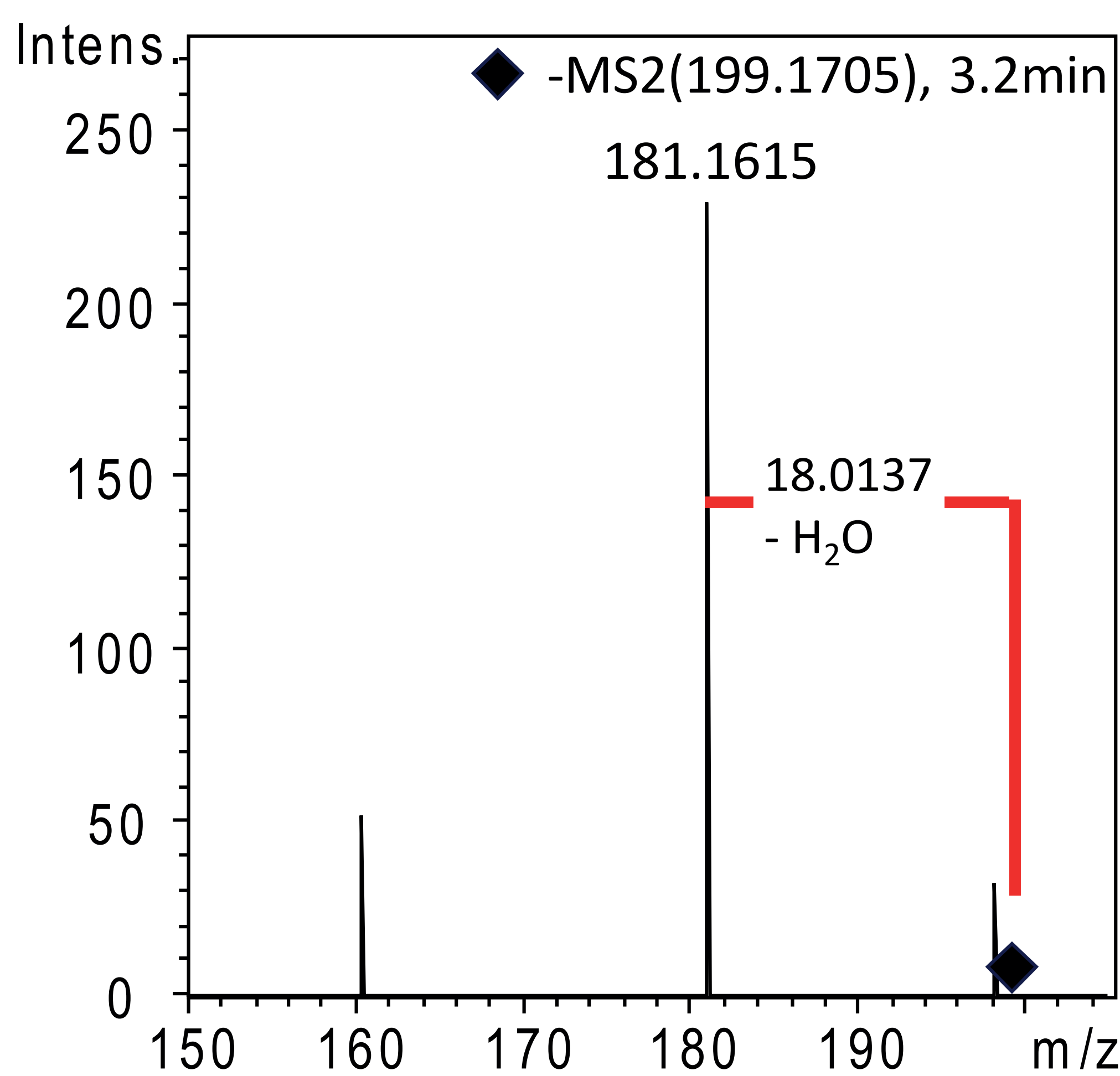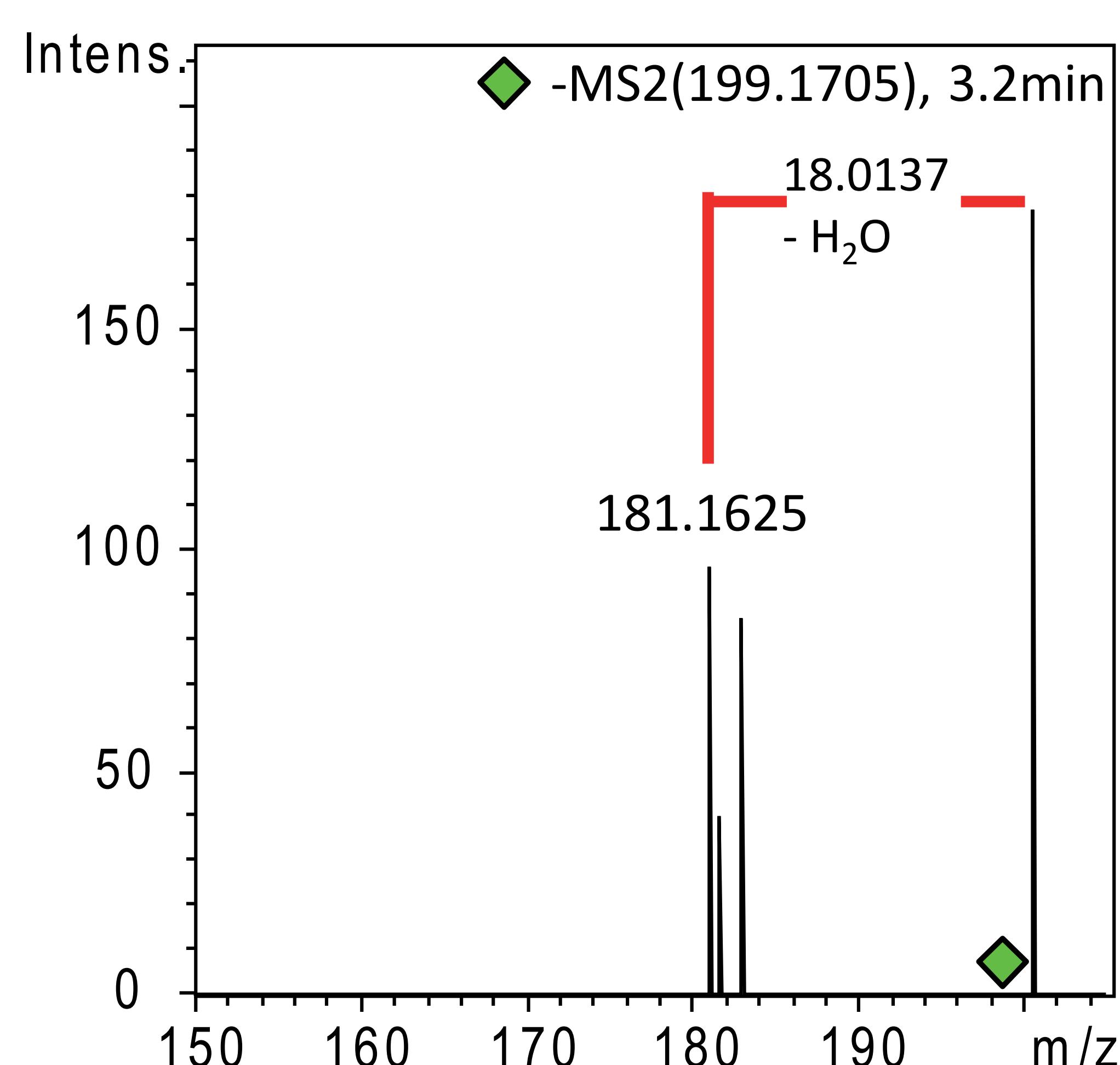**B**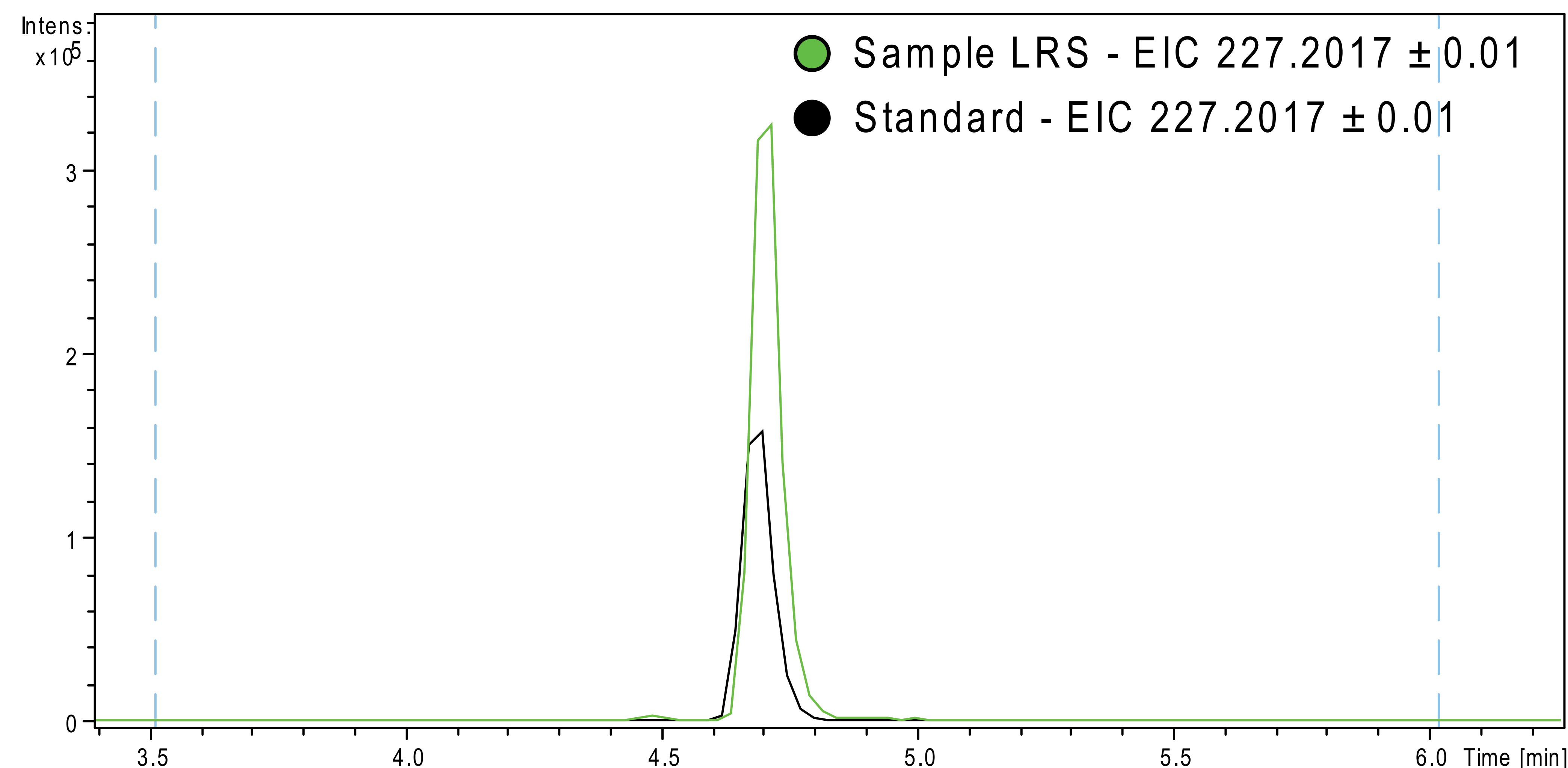**UPLC-QToF-MS**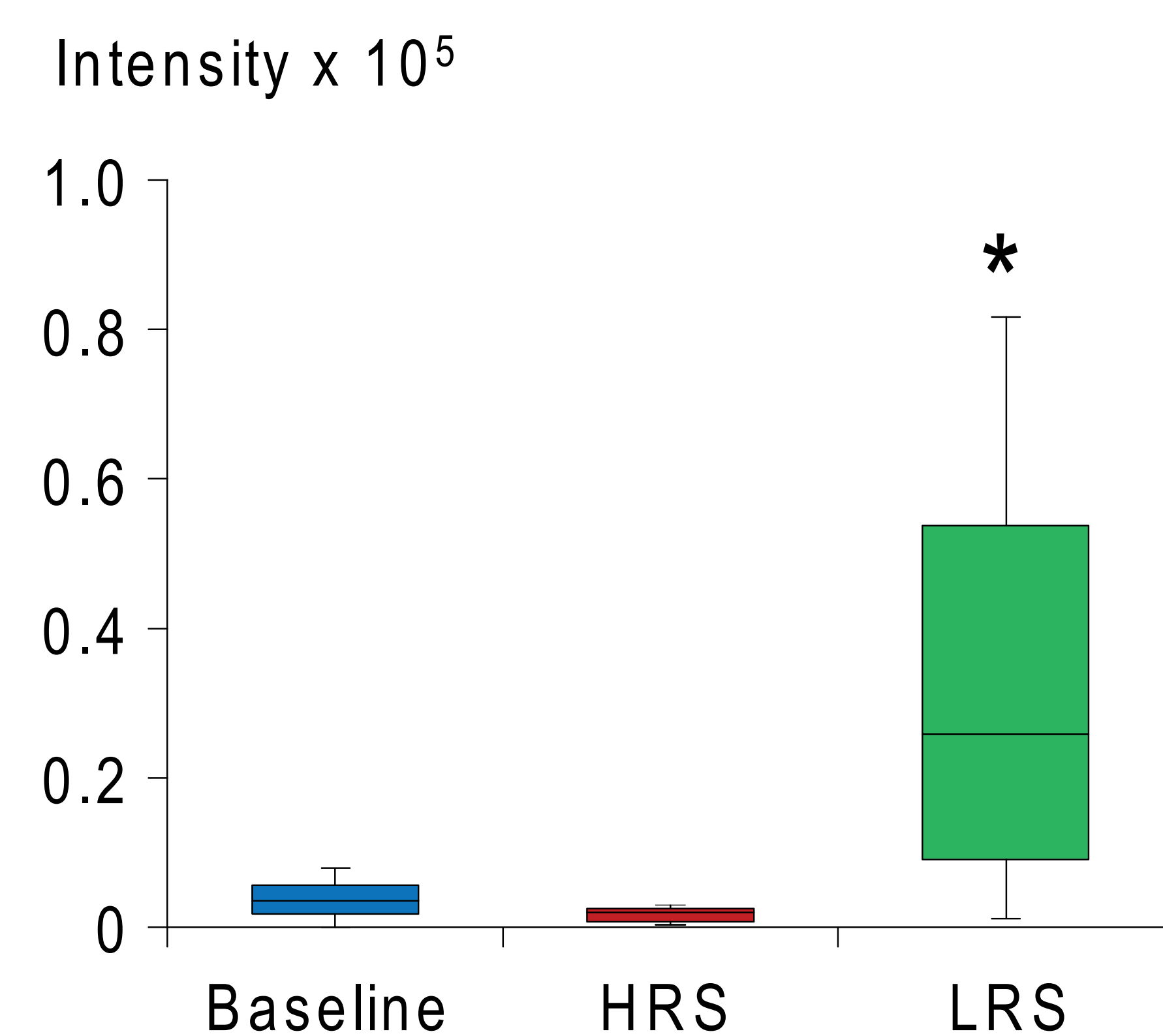**FT-ICR-MS**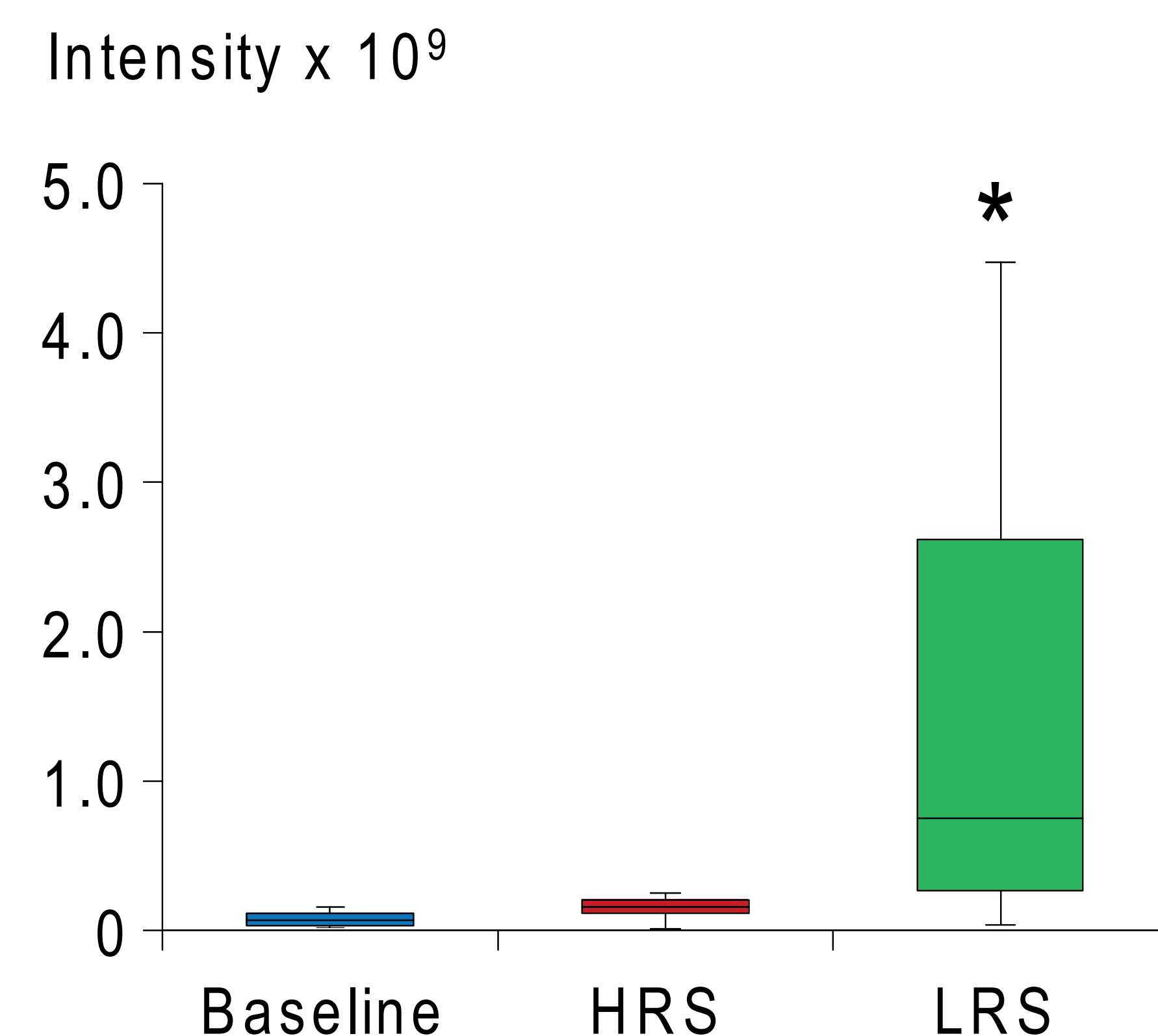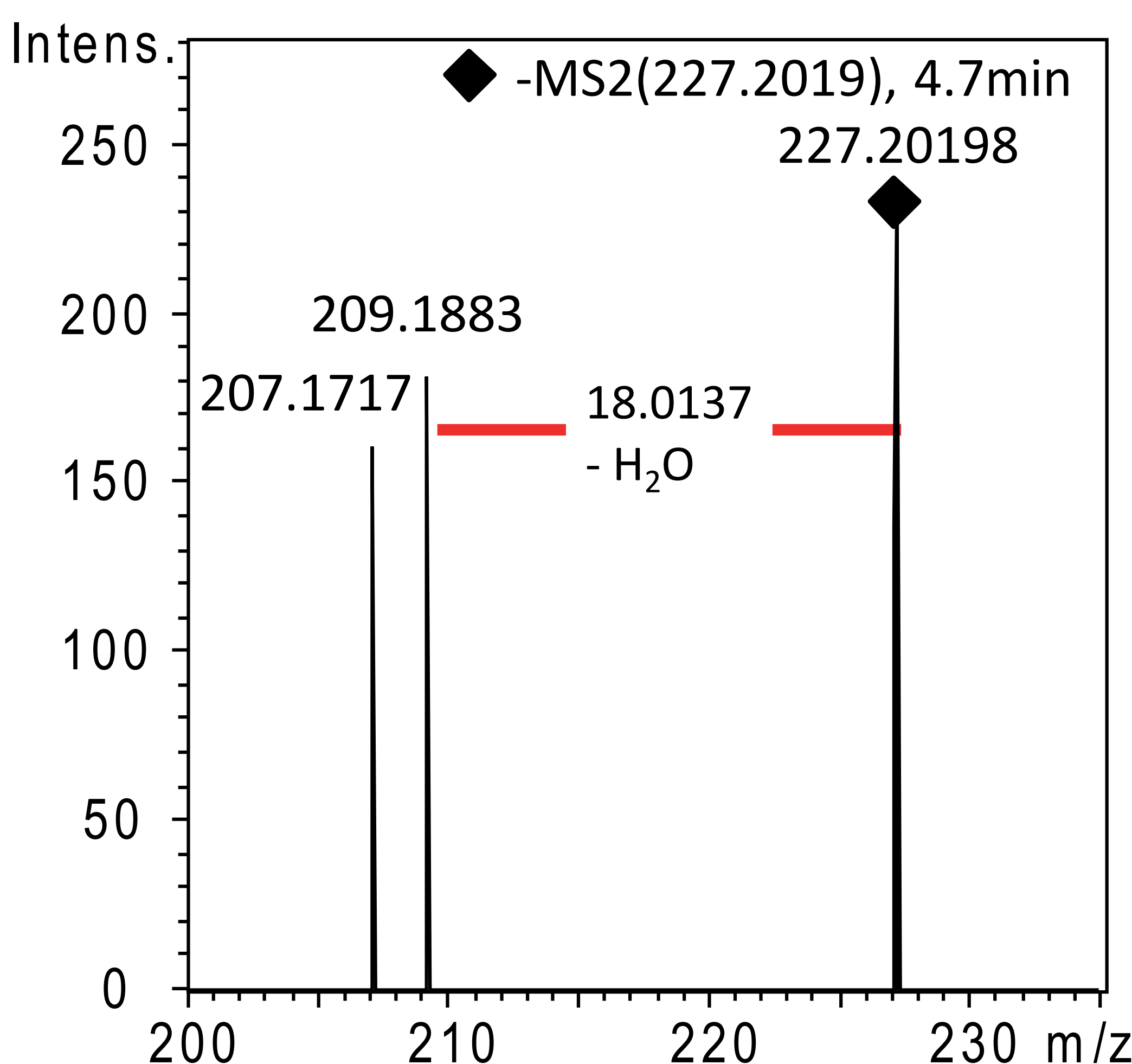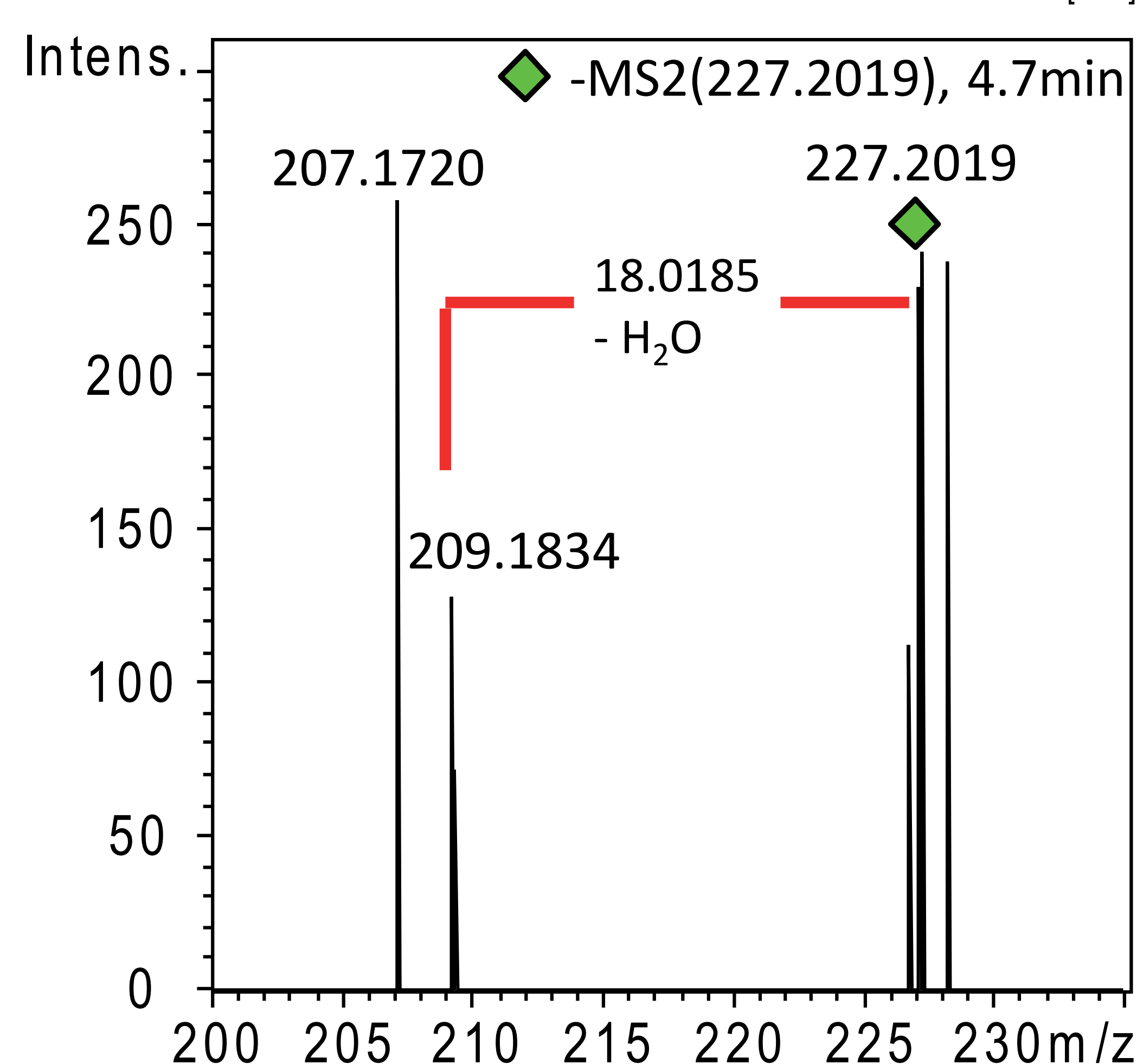

Supplement: FIG S6 [file mbo001173530sf6.pdf]

PC 2 (10.17%)

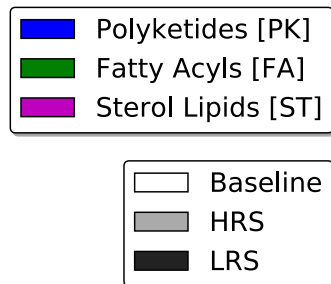

PC 1 (12.71%)

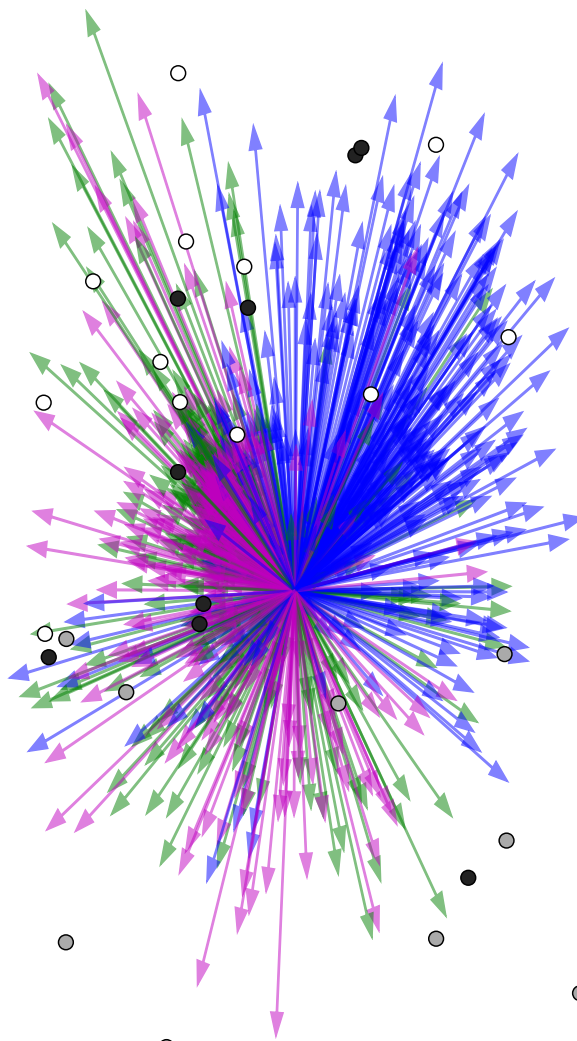

Supplement: FIG S7 [file mbo001173530sf7.pdf]

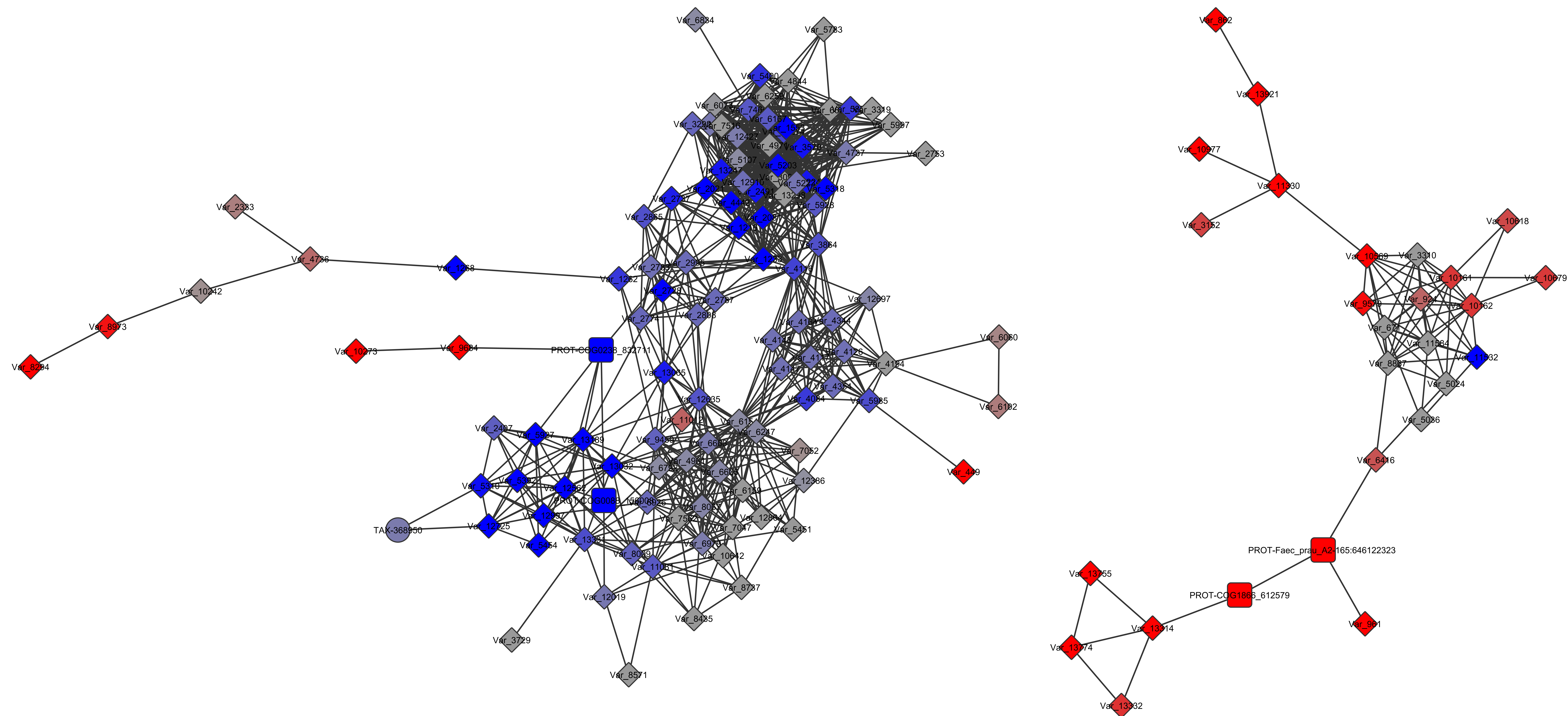

Supplement: FIG S8 [file mbo001173530sf8.pdf]
