# Supplementary material for: Impact of Dietary Resistant Starch on the Human Gut Microbiome, Metaproteome, and Metabolome
Source: mBio. 2017 Oct 17;8(5):e01343-17. doi: 10.1128/mBio.01343-17 (PMC5646248; doi:10.1128/mBio.01343-17)
Supplement: FIG S3 [file mbo001173530sf3.pdf]

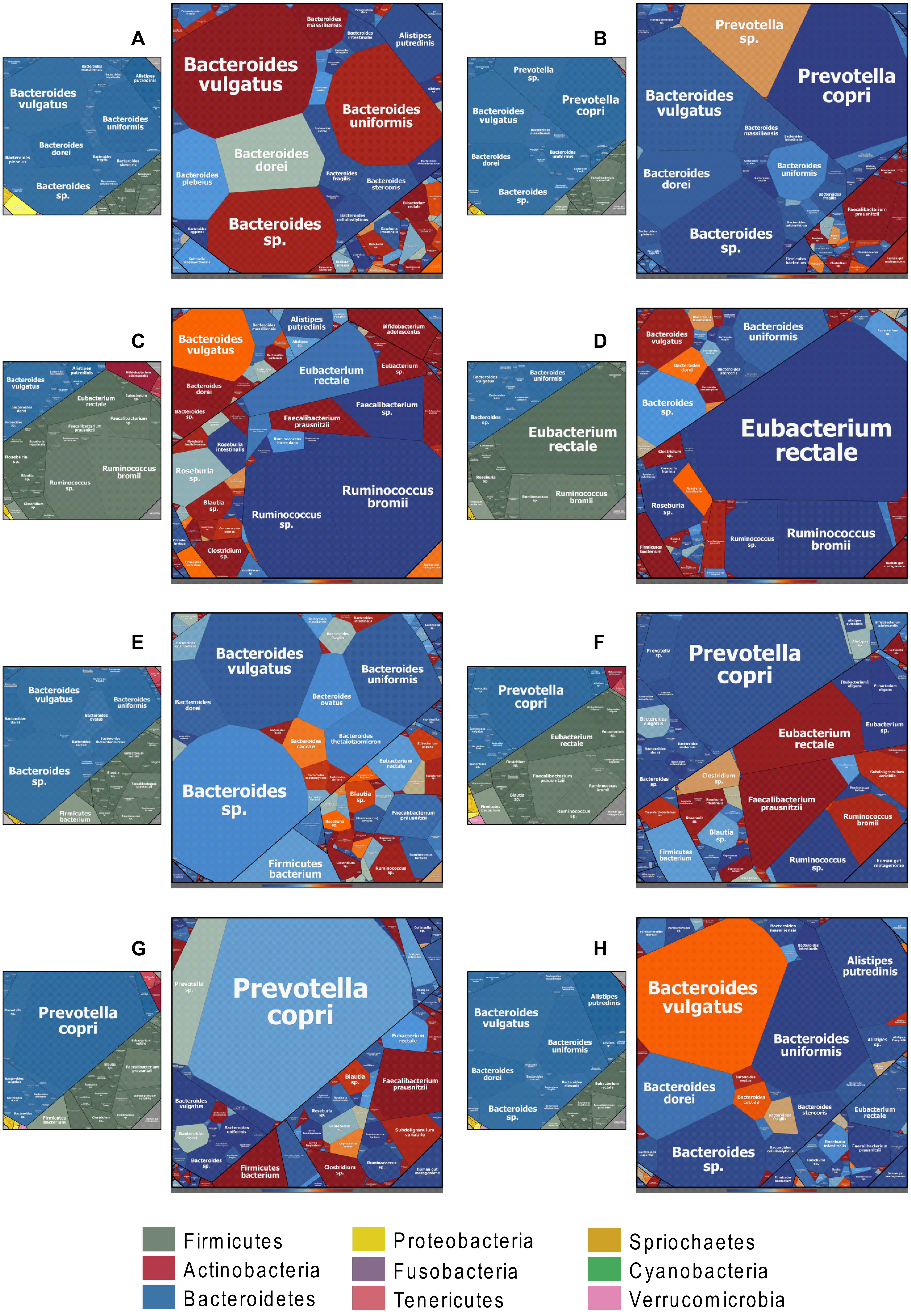
A
Bacteroides vulgatus
Bacteroides uniformis
Bacteroides dorei
Bacteroides sp.
Bacteroides plebeius
Bacteroides fragilis
Bacteroides stercoris
Bacteroides massiliensis
Bacteroides intestinalis
Alistipes putredinis
B
Prevotella sp.
Prevotella copri
Bacteroides vulgatus
Bacteroides dorei
Bacteroides uniformis
Bacteroides sp.
Bacteroides massiliensis
Faecalibacterium prausnitzii
C
Bacteroides vulgatus
Alistipes putredinis
Bifidobacterium adolescentis
Eubacterium rectale
Eubacterium sp.
Faecalibacterium sp.
Faecalibacterium prausnitzii
Roseburia sp.
Roseburia intestinalis
Ruminococcus bromii
Ruminococcus sp.
Blautia sp.
Clostridium sp.
D
Bacteroides vulgatus
Bacteroides uniformis
Bacteroides sp.
Eubacterium rectale
Ruminococcus bromii
Ruminococcus sp.
Roseburia sp.
Firmicutes bacterium
E
Bacteroides vulgatus
Bacteroides uniformis
Bacteroides ovatus
Bacteroides dorei
Bacteroides caccae
Bacteroides thetaiotaomicron
Bacteroides sp.
Eubacterium rectale
Faecalibacterium prausnitzii
Blautia sp.
Firmicutes bacterium
F
Prevotella copri
Prevotella sp.
Eubacterium rectale
Eubacterium sp.
Clostridium sp.
Faecalibacterium prausnitzii
Subdoligranulum variabile
Ruminococcus bromii
Ruminococcus sp.
Blautia sp.
Firmicutes bacterium
human gut metagenome
G
Prevotella copri
Prevotella sp.
Eubacterium rectale
Blautia sp.
Faecalibacterium prausnitzii
Subdoligranulum variabile
Firmicutes bacterium
Clostridium sp.
Ruminococcus sp.
Bacteroides vulgatus
H
Bacteroides vulgatus
Alistipes putredinis
Bacteroides uniformis
Bacteroides dorei
Bacteroides caccae
Bacteroides stercoris
Eubacterium rectale
Bacteroides sp.
Firmicutes
Actinobacteria
Bacteroidetes
Proteobacteria
Fusobacteria
Tenericutes
Spriochaetes
Cyanobacteria
Verrucomicrobia
