# Supplementary material for: Impact of Dietary Resistant Starch on the Human Gut Microbiome, Metaproteome, and Metabolome
Source: mBio. 2017 Oct 17;8(5):e01343-17. doi: 10.1128/mBio.01343-17 (PMC5646248; doi:10.1128/mBio.01343-17)
Supplement: FIG S5 [file mbo001173530sf5.pdf]

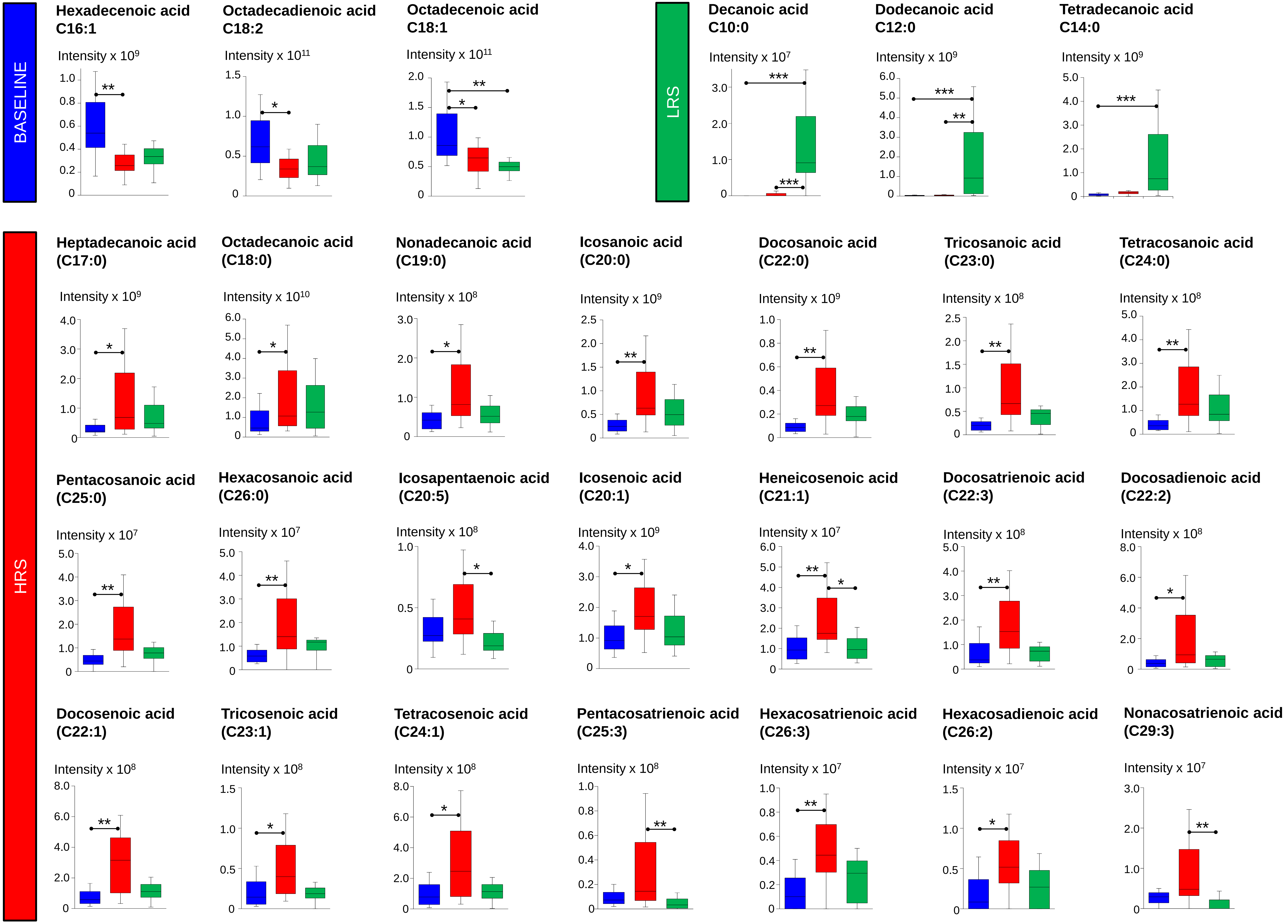

Level of Significance (p-value): 0 '***' 0.001 '**' 0.01 '*' 0.05
